# Investigation of Nrf2, AhR and ATF4 Activation in Toxicogenomic Databases

**DOI:** 10.3389/fgene.2018.00429

**Published:** 2018-10-02

**Authors:** Elias Zgheib, Alice Limonciel, Xiaoqi Jiang, Anja Wilmes, Steven Wink, Bob van de Water, Annette Kopp-Schneider, Frederic Y. Bois, Paul Jennings

**Affiliations:** ^1^Laboratoire de Biomécanique et Bio-ingénierie, Sorbonne Universités – Université de Technologie de Compiègne, Compiègne, France; ^2^Division of Molecular and Computational Toxicology, Amsterdam Institute for Molecules, Medicines and Systems, Vrije Universiteit Amsterdam, Amsterdam, Netherlands; ^3^Division of Biostatistics, German Cancer Research Center, Heidelberg, Germany; ^4^Division of Drug Discovery and Safety, Leiden Cell Observatory High Content Imaging Screening Facility, Leiden Academic Center for Drug Research, Leiden University, Leiden, Netherlands; ^5^Models for Ecotoxicology and Toxicology Unit (DRC/VIVA/METO), Institut National de l'Environnement Industriel et des Risques, Verneuil-en-Halatte, France

**Keywords:** transcriptomics, Nrf2, AhR, ATF4, toxicity pathways, toxicogenomic, oxidative stress

## Abstract

Toxicological responses to chemical insult are largely regulated by transcriptionally activated pathways that may be independent, correlated and partially or fully overlapping. Investigating the dynamics of the interactions between stress responsive transcription factors from toxicogenomic data and defining the signature of each of them is an additional step toward a system level understanding of perturbation driven mechanisms. To this end, we investigated the segregation of the genes belonging to the three following transcriptionally regulated pathways: the AhR pathway, the Nrf2 pathway and the ATF4 pathway. Toxicogenomic datasets from three projects (carcinoGENOMICS, Predict-IV and TG-GATEs) obtained in various experimental conditions (in human and rat *in vitro* liver and kidney models and rat *in vivo*, with bolus administration and with repeated doses) were combined and consolidated where overlaps between datasets existed. A bioinformatic analysis was performed to refine pathways' signatures and to create chemical activation capacity scores to classify chemicals by their potency and selectivity of activation of each pathway. With some refinement such an approach may improve chemical safety classification and allow biological read across on a pathway level.

## Introduction

Many transcriptionally activated pathways are intimately involved in responses to chemical induced perturbations and toxicological outcomes (Jennings et al., 2013). Here we focus on three such pathways. (1) The Nrf2 pathway (Nuclear Factor (Erythroid-derived 2)-Like 2 NFE2L2) which regulates the response to oxidative stress, (2) the ATF4 (Activating Transcription Factor 4) branch of the unfolded protein response and (3) the dioxin response or AhR pathway (Aryl Hydrocarbon Receptor). While these pathways have specific non-overlapping activation mechanisms and specific non-overlapping DNA binding elements reviewed in (Jennings et al., 2013), they also have overlapping downstream target genes. Adding to this complexity, converging toxicological mechanisms may lead to co-activation.

Oxidative stress is a major cause of chemical-induced injury and associated chronic diseases (e.g., cancer or Parkinson's disease) (Taguchi et al., 2011; Kong et al., 2014). The Nrf2 pathway the main adaptive response to oxidative stress. The Nrf2 protein exists in an inactive, cytoplasm-localized state, that is bound to the cytoskeleton-associated KEAP1 which facilitates Nrf2 ubiquitination and degradation. Upon oxidative stress, a conformational change in KEAP1 makes its binding to Nrf2 less favorable. Nrf2 stabilizes and translocates to the nucleus where it binds the antioxidant response element and drives the transcription of a genes involved in glutathione synthesis and recycling, xenobiotic metabolism and transport, and antioxidant genes (Jennings et al., 2013). ATF4 is a major branch of the unfolded protein response and is activated in response to endoplasmic reticulum (ER) disturbances or proteotoxicity where unfolded proteins accumulate in the ER and compete with PERK for the inhibitory protein BiP (Leonard et al., 2014). Activated PERK phosphorylates eIF2a which inhibits general protein translation while inducing AT4 translation. ATF4 in turn binds to the CARE consensus sequence and drives transcription of genes involved in amino acid synthesis, amino acid transport and aminoacyl-tRNA synthesis (Leonard et al., 2014). Xenobiotics can also activate specific genes through the AhR pathway. Upon ligand (xenobiotic) binding, the AhR transcription factor (TF) shuttles into the nucleus where it dimerizes with the “AhR nuclear translocator” and binds to so-called xenobiotic-responsive elements (XRE), aka dioxin response element (DRE), in the promoter region down stream targets including cytochrome P1-450 A1 (*CYP1A1*) (Haarmann-Stemmann et al., 2012).

Measuring the activation of transcriptionally regulated pathways such Nrf2, AhR, and ATF4 using transcriptomic approaches has great potential in increasing mechanistic understanding of chemical perturbations and to develop better prediction tools (Aschauer et al., 2015; Limonciel et al., 2015). Also, such an approach could be used for biological read across. However, there is still a knowledge gap pertaining to the interplay between the Nrf2, AhR, and ATF4 pathways. It is known that several of their downstream targets have promotor sequences for more than one of these TFs. For example, NQO1 is driven by both AhR and Nrf2. Also, it is likely that the pathways may cooperate in redressing certain hoemeostatic perturbations. For example, we have shown that Nrf2 and ATF4 cooperate on the level of glutathione, where ATF4 promotes the uptake of glutathione amino acid building blocks including glutamine and cysteine and promotes glutamate production via induction of asparagine synthetase. Nrf2 in turn through induction of glutamate cysteine ligase and glutathione synthase produce new glutathione (Wilmes et al., 2013). Very little is known about species differences, tissue specificity, chemical specificity, or other subtleties in the activation of these pathways.

To investigate this further, we performed a transcriptomic analysis of large and medium size toxicogenomic datasets from the EU 6th and 7th framework projects carcinoGENOMICS and Predict-IV, as well as from TG-GATEs. Within these studies we also identified some potentially useful specific activators of the pathways investigated. Potassium bromate and phorone have been used to experimentally activate Nrf2. Potassium bromate is an oxidizing agent causing ROS injury and oxidative stress-induced DNA damage (Ballmaier and Epe, 1995; Limonciel et al., 2012). In a recent study we showed that potassium bromate activated the Nrf2 and p53 response without activation of the ATF4 response (Limonciel et al., 2018). Phorone can similarly activate Nrf2 due to glutathione depletion (Younes et al., 1986; Iannone et al., 1990; Oguro et al., 1996). Tunicamycin is a prototypical activator of the unfolded protein response (including the ATF4 branch) by causing an accumulation of misfolded glycoproteins in the ER (Oslowski and Urano, 2011). More specifically, tunicamycin inhibits the N-glycosylation of newly formed proteins by DPAGT1, leading to an interruption in glycoprotein production (Bassik and Kampmann, 2011). Benzo(a)pyrene and omeprazole have been used to activate AhR. Benzo(a)pyrene is a polycyclic aromatic hydrocarbon and a prototypical AhR agonist (Nebert et al., 2004). Omeprazole, a proton pump inhibitor (Howden, 1991, 199) is also an AhR activator (Jin et al., 2012, 2014).

The aim of the investigation was to investigate potential co-dependences of ATF4, Nrf2 and/or AhR, to develop a signature panel for each pathway and to develop a chemical activity scoring system, for chemical grouping.

## Materials and methods

### Generation of target gene lists

For each of the three TF of interest (AhR, Nrf2, and ATF4), the following three search strategies, from the works of (Limonciel et al., 2015), were applied in PubMed to retrieve TF target genes: (i) search for TF name and ChIP-sequencing or ChIP-microarray studies, (ii) search for TF name and TF-specific response element and “Electrophilic Mobility Shift Assay” or ChIP studies, and (iii) search for TF name and TF-specific DNA response element and name of a target gene known. In the first tier of this strategy, high-throughput sequencing datasets were retrieved, which provided extensive lists of genes shown to have the TF bind in their promoter region. In the second tier, lower throughput investigations were included, providing target genes that were more deeply investigated in the article with proven TF binding of the promoter region. These first two tiers provided an unbiased source of target genes that was completed in the third tier with manually added target genes for which at least one study showed binding of the TF in their promoter region.

PubMed searches were performed on 24.11.2014 for Nrf2 and 17.12.2014 for ATF4 and AhR. Gene lists are reported in Supplementary Table 1 and illustrated on Supplementary Figure 1.

### Construction of a chemical-effects transcriptomics database

The database of chemical-induced transcriptomic changes comes from three projects: carcinoGENOMICS (Vinken et al., 2008), Predict-IV (Mueller et al., 2015) and TG-GATEs (Igarashi et al., 2015). In carcinoGENOMICS, human and rat kidney cells were exposed to bolus concentrations of up to 31 chemicals in *in vitro* settings for up to 72 h. In Predict-IV, human kidney cells and liver cells from human and rat were exposed daily *in vitro* for up to 14 days to up to 22 chemicals. Up to 171 chemicals from TG-GATEs were tested in various rat *in vivo* and *in vitro systems*, with various treating regimes. Table 1 summarizes this and shows the 211 chemicals tested and dispatched in different categories of one or more of the three projects. Supplementary Table 2 presents the exhaustive lists of chemicals by category.

**Table 1:** Number of chemicals used in each experimental category.

**Project**	**Species**	**Tissue**	**Setting**	**Mode**	**Time-points**	**Number of chemicals**	**Notes**
All dataset [211]^*^							(1–2)
Carcino-GENOMICS [31]	Human	Kidney	*in vitro*	Bolus	6h, 24h, 72h	30	(3–4)
	Rat	Kidney	*in vitro*	Bolus	6h, 24h, 72h	15	
PREDICT-IV [22]	Human	Kidney	*in vitro*	Repeated doses	1d, 3d, 14d	12	(5–6)
	Human and Rat	Liver	*in vitro*	Repeated doses	1d, 3d, 14d	11	(7)
TG-GATEs [171]	Human	Liver	*in vitro*	Bolus	2h, 8h, 24h	160	(8)
	Rat	Liver	*in vitro*	Bolus	2h, 8h, 24h	145	(9)
		Liver	*in vivo*	Bolus	3h, 6h, 9h, 24h	158	(10–11)
		Liver	*in vivo*	Repeated doses	4d, 8d, 15d, 29d	143	–
		Kidney	*in vivo*	Bolus	3h, 6h, 9h, 24h	41	(12)
		Kidney	*in vivo*	Repeated doses	4d, 8d, 15d, 29d	41	

(1) Number of chemicals assayed in at least one of the three source projects.

(2) **Cyclosporine A** is the only chemical that was used in the three projects. **Cyclosporine A** appears in every single experimental category and sub-category (except carcinoGENOMICS's Rat tests).

(3) In carcinoGENOMICS, all 15 chemicals tested on rat cells, except one (**Dimethylnitrosamine**), were also tested on human cells.

(4) Beside **Cyclosporine A**, and five of the chemicals that appear in TG-GATEs as well, all chemicals are specific to carcinoGENOMICS [**2-Nitrofluorene** and **N-nitrosomorpholine** (TG-GATEs “Human liver in vitro bolus” and “Rat liver in vivo bolus”); and **Diclofenac**, **Nifedipine** and **Tolbutamide** (all liver categories of TG-GATEs)].

(5) The 12 chemicals tested on kidney cells and the 11 tested on liver cells in PREDICT-IV are distinct; Only **Cyclosporine A** is presented in these two categories.

(6) Among the chemicals tested on kidney cells in PREDICT-IV, only **Cisplatin** appears elsewhere (in TG-GATEs rat tests).

(7) Among the chemicals tested on liver cells in PREDICT-IV, only **Acetaminophen** and **Valproic acid** appear in all TG-GATEs categories; **Amiodarone**, **Chlorpromazine**, **Fenofibrate**, **Ibuprofen** and **Metformin** were tested on liver cells of TG-GATEs, and **Rosiglitazone** as well (except in “Rat liver in vitro bolus”).

(8) In TG-GATEs, five chemicals were tested on human cells only (**HGF**, **IL1beta**, **IL6**, **INFalpha**, **Nefazodone**, and **TGFbeta1**) and six others on animal categories only (**Carboplatin**, **Cephalotin**, **Cisplatin**, **Gentamicin**, **TNFalpha**, and **Trimethadione**).

(9) Five chemicals appear in liver in vitro bolus categories only (human and rat): **Alpidem**, **Buspirone**, **Clozapine**, **Nefazodone** and **Venlafaxine**.

(10) **3-Methylcholantrene**, **Bortezomib**, **Gefitinib**, **Imatinib**, and **Puromycin** appear in the “Rat liver in vivo bolus” category exclusively.

(11) **2-Nitrofluorene**, **Aflatoxin B1**, **Dexamethasone**, **N-methyl-N-nitrosourea** and **TNF** are common to TG-GATEs' “Human” and “Rat liver in vivo bolus” categories and were not tested in other conditions.

(12) The 41 chemicals that are used for TG-GATEs kidney in vivo testing are the same for both modes (bolus and repeated doses) and are common for all other categories (exceptions: **Gentamicin**, **Carboplatin**, **Cephalotin**, **Cisplatin**, **Desmopressin acetate**, **Amphotricine B**, and **Acetamide)**.

* *The number between brackets refers to the number of chemicals per project*.

#### Data sources

The carcinoGENOMICs and Predict-IV data are publicly accessible on the diXa database hosted by The European Bioinformatics Institute^1^. In carcinoGENOMICS, *in vitro* renal cell experiments were performed using the human cell lines RPTEC/TERT1 (human, telomerase transfected) and NRK-52E (rat). The study no. is DIXA-003. Differentiated cell cultures were exposed to a single bolus of non or low cytotoxic (<IC10) concentration of chemical for 6, 24, or 72 h before lysis in TRIZOL, RNA purification and transcriptomic analysis on Affymetrix microarrays as described (Limonciel et al., 2012). Affymetrix Human Genome U133 Plus 2.0 GeneChIP arrays were used for human samples and Rat Genome 230 2.0 GeneChIP for rat samples. Normalization quality controls, including scaling factors, average intensities, present calls, background intensities, noise and raw Q-values were within acceptable limits for all chips. Hybridization controls were identified on all chips and yielded the expected increases in intensities. All subsequent analyses were based on normalized expression values generated using the MAS5 normalization algorithm. It is noted that RMA or GCRMA normalization would have been preferred. Normalized data was imported into GeneSpring (Agilent) to identify log2 fold change values for selected genes.

Within PREDICT-IV, *in vitro* testing of nephrotoxic and hepatotoxic compounds were performed on RPTEC/TERT1 cells (renal model), primary human hepatocytes, and rat hepatocytes (PHH and PRH, respectively). The study no. on the diXa database is DIXA-095. Differentiated cell cultures were exposed daily to a high (≤10% cell death) or low concentration of chemical for 1, 3 or 14 days, as described (Wilmes et al., 2013, 2014; Aschauer et al., 2015; Crean et al., 2015; Limonciel et al., 2015). Transcriptomic analysis was carried out on Illumina® HT 12 v4 BeadChip arrays for kidney and PHH human samples, except RPTEC/TERT1 exposed to CsA (HT 12 v3 chips). PRH samples were analyzed with Illumina® RatRef-12 v1 BeadChIP arrays. Results were normalized by quantile normalization and expressed as log2 fold over time-matched control. Where several probes existed for a given gene, the probe with the highest variation across the dataset was selected.

The TG-GATEs datasets comprised *in vivo* rat data from liver and kidney tissue, as well as data from *in vitro* primary rat and human hepatocyte cultures, after a single administration of chemical and repeat dosing (see Table 1)^2^. CEL files were downloaded from the Open TG-GATEs database of the Toxicogenomics Project and Toxicogenomics Informatics Project under CC Attribution-Share Alike 2.1 Japan. Probe annotation for the primary human hepatocyte data was performed using the hthgu133pluspmhsentrezg.db package version 17.1.0 and probe mapping was performed with hthgu133pluspmhsentrezgcdf downloaded from NuGO^3^. Probe annotation for the rat data was performed using the rat2302rnentrezg.db package version 19.0.0 and probe mapping was performed with the rat2302rnentrezgcdf package version 19.0.0 downloaded from NuGO. These mappings summarize the corresponding probes to a single probe set per gene. Probe-wise background correction (Robust Multi-Array Average expression measure), between-array normalization within each treatment group (quantile normalization) and probe set summaries (median polish algorithm) were calculated with the RMA function of the Affy package (Affy package, version 1.38.1) (Irizarry et al., 2003). The normalized data were statistically analyzed for differential gene expression using a linear model with coefficients for each experimental group within a treatment group (Wolfinger et al., 2001). A contrast analysis was applied to compare each exposure with the corresponding vehicle control. For hypothesis testing the moderated t-statistics by empirical Bayes moderation was used followed by an implementation of the multiple testing correction of Benjamini and Hochberg (Hochberg and Benjamini, 1990) using the LIMMA package (Smyth et al., 2005).

All interspecies gene conversions where done using the provided human gene symbols which were converted to human or rat gene identifiers using the online conversion tool of bioDBnet^4^.

Altogether, the collected data concern 804 genes from the 857 genes identified in PubMed as targets of AhR, Nrf2 and ATF4. The 53 target genes that are not covered with data from any of the three projects were excluded from this study. These genes are listed in the last row of Supplementary Table 1.

### Bioinformatics methods

#### Data selection

The heterogeneity of the sources of information of our database widens its coverage and strengthens its capacity to represent multiple conditions. However, this richness makes the database's structure complex. To simplify the analysis without losing potentially important information, we focused on conditions providing the best background to study the three pathways individually. The effects observed following exposure to a chemical could vary greatly depending on exposure duration. Exposures lasting more than 24 h tend to cause mixed stress responses that make it difficult to delineate the activation of specific molecular pathways and the initial mechanisms of toxicity of chemicals. These conditions could be a potential source of noise for the analysis and were thus excluded. Excluding all data obtained after 24 h reduced the dataset from 7,042 to 4,685 testing conditions. We chose not to eliminate the early kidney *in vivo* time points (at 3 and 6 h), even though they may be more reflective of background levels in case of slow absorption of the chemical administered.

#### Pathway specific chemicals

In order to distribute the genes to pathways and pathway overlapping zones, log2 genes fold changes (FC) were ranked in decreasing order and examined on reduced datasets containing conditions relative to pathway specific activators. We define a pathway specific activator as a chemical where the mode of action is known, that the mode of action activates the specific pathways and that this mode of action is not expected to activate the other pathways under investigation. Thus, at relatively short exposures, to relatively low concentrations these chemicals will only act on their specific target. It is however possible at higher concentrations or longer time exposure, other targets will be affected due to increasing toxicity. As shown in Table 1, some chemicals were not tested in all categories and tissue types. Thus, it was not possible to find pathway specific activators able to cover the entire database. Table 2 shows the coverage of the datasets by the pathway specific activators selected as reference for analysis. Although none of the toxicogenomic databases analyzed here were designed to specifically address any of our three pathways of interest, most datasets included at least one chemical that could be considered as a specific pathway activator. Two specific chemicals were selected for AhR (Benzo(a)pyrene and Omeprazole) and Nrf2 (Potassium Bromate and Phorone) and one for ATF4 (Tunicamycin). However, within “Rat Kidney *in vivo*” category, no Nrf2 specific chemicals were found, and for all kidney data no ATF4 specific chemical were found either.

**Table 2:** Chosen pathway specific chemical through the dataset.

**Pathway**	**Species**	**Kidney**		**Liver**	
		***in vitro***	***in vivo***	***in vitro***	***in vivo***
AhR	Human	Benzo(a)pyrene		Omeprazole	
	Rat				
Nrf2	Human	Potassium Bromate		Phorone	
	Rat				
ATF4	Human			Tunicamycin	
	Rat				

#### Construction of pathway signatures

For each of the pathway specific chemicals, all testing conditions were selected. For every gene, the mean of log2(FC) throughout all those conditions was calculated, to form the average activation value of each gene by each of the pathway specific activator. For AhR and Nrf2, the two average activation values obtained (one for each of the pathway specific activator) were themselves averaged. Genes were then sorted in decreasing order of average activation values per pathway. It is important to note that, since the expression of some genes can be inhibited (down regulated) by some chemicals or in certain conditions, some of the average activation values were negative. In order to select the most sensitive genes for each pathway, we computed the mean (μ) and the standard deviation (σ) of the genes' average activation values in each list. A pathways signature was formed by the genes whose average activation values were greater than μ + 2σ or smaller than μ – 2σ for this pathway. Genes appearing in the signature of more than one pathway were set apart in “overlapping signatures.”

Furthermore, we stratified signatures by original databases' categories (“Rat liver cells *in vitro*,” “Rat liver cells *in vivo*,” “Human liver cells *in vitro*” etc.) (which correspond to primary cells), to check if there would be any species-specific or *in vitro*/*in vivo* differences among signatures. We chose to work only with liver data since more data were available for liver (602 conditions in kidney vs. 4,083 tested in liver, see Table 3). Following the same procedure as above, we constructed pathway signatures for AhR, Nrf2, and ATF4 in each of the following liver categories: (a) Rat liver cells *in vitro*, (b) Rat liver cells *in vivo*, and (c) Human liver cells *in vitro*.

**Table 3:** Number of conditions (chemicals, concentrations, time-points) tested per category.

**Pathway**	**Species**	**Kidney**		**Liver**		**TOTAL**
		***in vitro***	***in vivo***	***in vitro***	***in vivo***	
Human		85	0	963	0	1048
Rat		30	487	1282	1838	3637
Total		602		4083		4685

In all cases, general or stratified, some genes were excluded for having no data on effect of the chosen pathway specific chemicals. A list of those genes appears in Supplementary Table 3.

**Figure 1:**
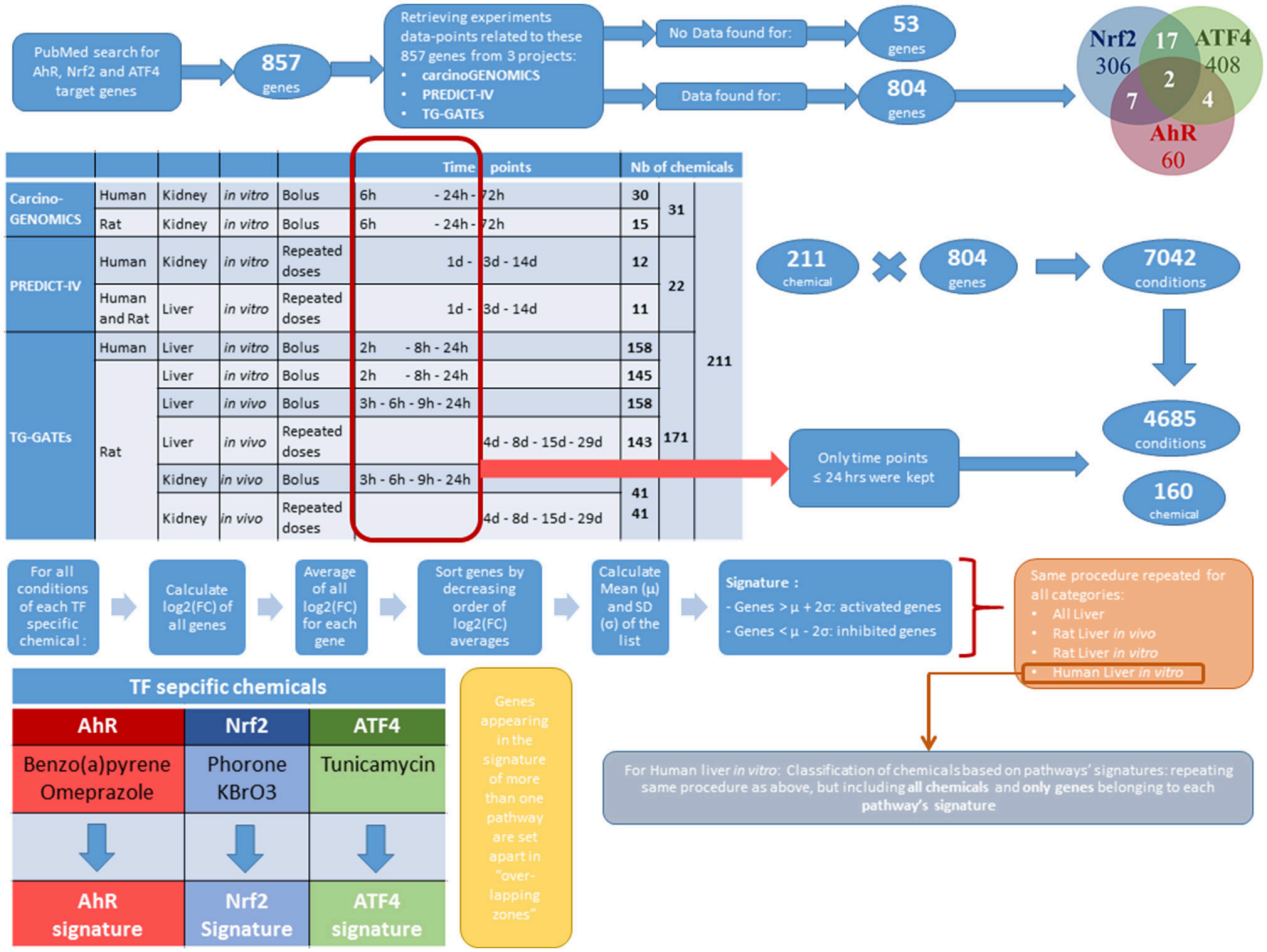
Methods summarizing workflow.

A summary of the above-described protocols and the following procedures of Methods are presented in the workflow of Figure 1.

#### Pathway's signature-based prioritization of chemicals

Among the three liver categories where signatures were stratified, we chose to focus on the “Human liver cells *in vitro*” sub-category exclusively since the ultimate goal of our toxicity pathways' analyses and models is risk assessment of human cells' exposure to xenobiotics. We considered only the genes belonging to the signature of each of the three pathways, but not their overlapping zones. This selection of experimental category and genes reduces the number of studied chemicals from 211 to 160 for the lack of data on the rest of chemicals in this section. Then, for each of the 160 chemicals investigated, we averaged log2(FC) of the pathway signature genes over experimental conditions. Therefore, for each of the three pathways, we obtained a “chemical activation capacity” (CAC) value per chemical. This value reflects how strongly a chemical can activate a given toxicity pathway. Those CACs can be negative for chemicals inhibiting the majority of the genes of a pathway. We used CACs to estimate the pathway's selectivity of chemicals as well as the importance of their impact.

**Figure 2:**
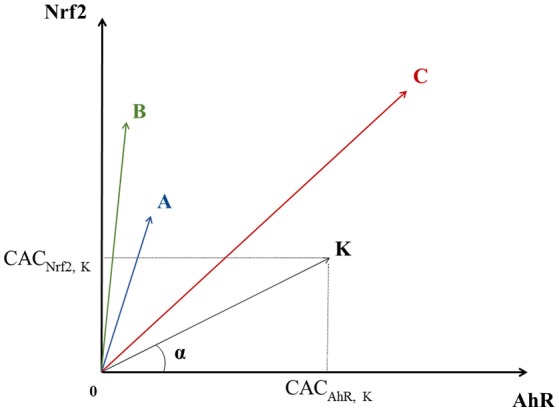
Geometric representation of chemical specificity and potency for the Nrf2 and AhR pathways. *K* represents a chemical and its coordinates are (CAC_AhR_, _K_, CAC_Nrf2_, _K_). *K* also defines the vector $\vec{OK}$ linking the origin *O* (0, 0) to point *K*. The absolute value of the cosine of the angle α between $\vec{OK}$ and a pathway's axis can be used to measure the specificity of a chemical for the given pathway (the smaller α, the more specific the chemical). On the other hand, the overall activation potency of a chemical increases proportionally with the length of $\vec{OK}$. Points *A, B*, and *C* represent three other chemicals with different specificities and potencies for pathways' activation (see text).

Each chemical can be considered as a point having three CACs as coordinates in a 3-dimensional space which axes correspond to a given pathway. Let us consider a chemical K that has a point in a bi-dimensional graph where the X-axis corresponds to AhR and the Y-axis to Nrf2. In this graph, K's coordinates would be: (CAC_AhR_, _K_, CAC_Nrf2_, _K_), see Figure 2. K also defines the vector $\vec{OK}$ linking the origin O (0, 0) to the point K.

The specificity of a chemical for a given pathway can be measured by the proximity of its point K to the axis representing that pathway. Proximity can be mathematically evaluated by the absolute value of the cosine of the angle (α) between the pathway's axis and $\vec{OK}$. The more K is specific to AhR, the closer it is to the AhR's axis, the smaller α is, and the bigger **cos** **(α)**. In theory, in a 3-dimensional space, a point is closer to an axis than to the two others when its **cos** **(α)** with this axis is greater than $\frac{1}{\sqrt{3}}$. Thus, the value of 0.57735 $\left(\frac{1}{\sqrt{3}}\right)$ was chosen as a cut-off point for **cos** **(α)**. On the other hand, the activation potency of a chemical proportionally increases with the module of the vector $\vec{OK}$ vector noted $\left||\vec{OK}|\right|$ (the distance between the origin and the chemical's point). The value of 0.5 was chosen as a cut-off point for $\left||\vec{OK}|\right|$. For instance, chemicals A and B in Figure 2 are both quite specific of Nrf2, but A's activation potency is relatively limited compared to B's $\left(\left||\vec{OA}|\right|<\left||\vec{OB}|\right|\right)$.

Similarly, even though C seems to have a greater activation potency than A and B (greater module), it is equidistant to both axes and therefore is not specific of any of the two pathways. The same logic applies for a 3-dimensional space, adding one extra axis for the ATF4 pathway.

In our signature-based classification of chemicals, for each pathway, after applying the chosen cut-off points, we sorted chemicals by the result of the product $\cos(\alpha )\times \;\left||\vec{OK}|\right|$. Thus, chemicals which are both pathway specific (high **cos** **(α)**) and potent $\left(high\left||\vec{OK}|\right|\right)$ show up first in our lists.

## Results

A visual depiction of the workflow is provided in Figure 1.

### Pathways' global signatures

**Figure 3:**
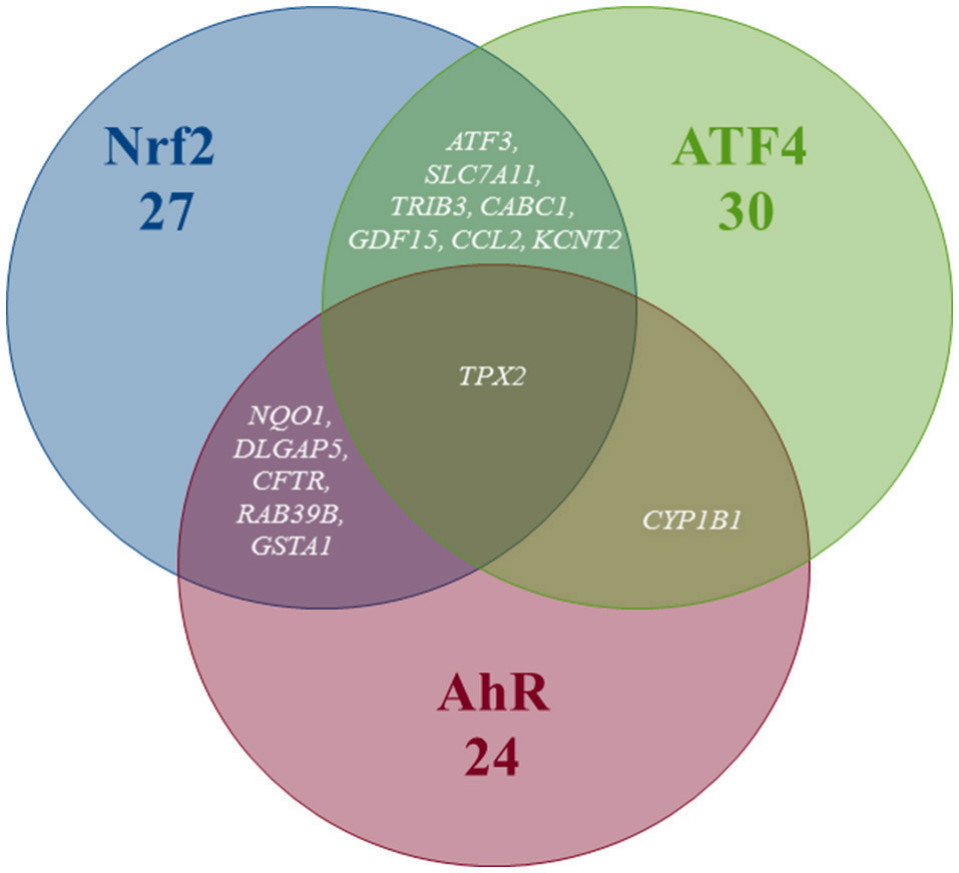
Venn diagram of the number of genes per pathway's global signatures and names of genes of overlapping zones.

**Figure 4:**
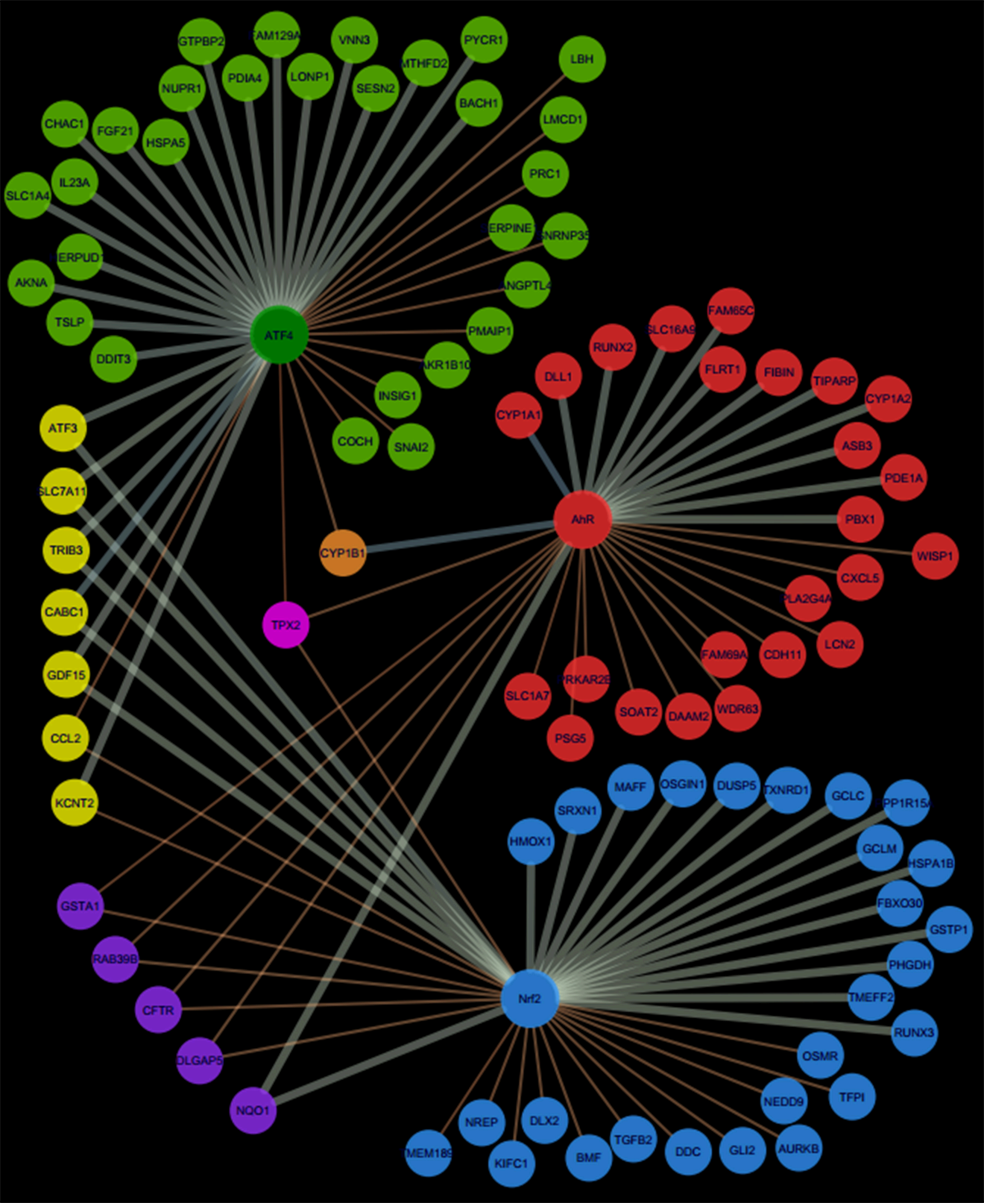
Network representation of AhR, Nrf2 and ATF4 pathway signatures and their overlapping zones.

Pathway's signatures defined on the basis of the whole data set are listed in Table 4. Each signature has two parts: “Activated genes” (those having positive log2(FC) averages and are greater than μ + 2σ) and “Inhibited genes” (those having negative log2(FC) averages and are smaller than μ – 2σ); The two parts are merged in one in the overlapping signatures. In all lists, genes are sorted by the decreasing absolute value of the genes' log2(FC) averages. The number of genes in the obtained pathway's signature was 24 for AhR, 27 for Nrf2 and 30 for ATF4. In each pathway, at least half (12 for AhR, 15 for Nrf2 and 19 for ATF4) were “Activated genes.” The *a priori* pathway is the one for which the gene has come up in PubMed searches; Table 4 shows that most of activated genes were *a priori* suspected to belong to the target pathway (for example: *CYP1A1, RUNX2*, and *CYP1A2* were known to be activated by AhR, *HMOX1* and *SRXN1* by Nrf2 and *DDIT3* and *HERPUD1* by ATF4; those genes are highlighted in gray) while this wasn't the case of the “Inhibited genes” part of the lists. Figure 3 shows the overlapping zones. Among the five genes that are in the AhR-Nrf2 overlapping zone (*NQO1, DLGAP5, CFTR, RAB39B* and *GSTA1*), only *NQO1* is a mainly activated gene while this was the case of most seven genes of the Nrf2-ATF4 overlapping zone (*ATF3, SLC7A11, TRIB3, CABC1, GDF15*) with two exceptions (*CCL2* has negative averages for both pathways and *KCNT2* for Nrf2). *CYP1B1* is the only mutual gene for AhR (strong activation) and ATF4 (inhibition) and *TPX2* is the only mutual gene for all three pathways (inhibition). Figure 4 shows a network representation of the three signatures and their overlapping zones.

**Table 4:** Pathways' global signatures for AhR, Nrf2 and ATF4 pathways and the signatures of their overlapping zones (AhR-Nrf2, Nrf2-ATF4, AhR-ATF4, and AhR-Nrf2-ATF4) for all available data.

**Activated genes**	**AhR Signature**			**Nrf2 Signature**			**ATF4 Signature**		
	**Genes**	**log_2_ (FC) averages**	***A priori* pathway**	**Genes**	**log_2_ (FC) averages**	***A priori* pathway**	**Genes**	**log_2_ (FC) averages**	***A priori* pathway**
	*CYP1A1*	4.35	AhR	*HMOX1*	1.12	Nrf2	*DDIT3*	1.59	ATF4
	*DLL1*	1.36	AhR	*SRXN1*	0.97	ATF4 Nrf2	*TSLP*	1.51	ATF4
	*RUNX2*	1.03	AhR	*MAFF*	0.78	AhR Nrf2	*AKNA*	1.30	ATF4
	*SLC16A9*	0.92	Nrf2	*OSGIN1*	0.67	Nrf2	*HERPUD1*	1.23	ATF4
	*FAM65C*	0.79	AhR	*DUSP5*	0.66	ATF4	*SLC1A4*	1.15	ATF4
	*FLRT1*	0.78	ATF4	*TXNRD1*	0.63	ATF4	*IL23A*	1.05	ATF4
	*FIBIN*	0.77	ATF4	*GCLC*	0.60	ATF4	*CHAC1*	0.99	ATF4
	*TIPARP*	0.73	AhR	*PPP1R15A*	0.57	ATF4	*FGF21*	0.95	ATF4
	*CYP1A2*	0.69	AhR	*GCLM*	0.57	Nrf2	*HSPA5*	0.94	ATF4
	*ASB3*	0.67	Nrf2	*HSPA1B*	0.56	Nrf2	*NUPR1*	0.94	ATF4
	*PDE1A*	0.66	ATF4	*FBXO30*	0.55	ATF4	*GTPBP2*	0.91	ATF4
	*PBX1*	0.64	Nrf2	*GSTP1*	0.53	Nrf2	*PDIA4*	0.87	Nrf2
				*PHGDH*	0.46	Nrf2	*FAM129A*	0.87	ATF4
				*TMEFF2*	0.46	ATF4	*LONP1*	0.80	ATF4
				*RUNX3*	0.46	Nrf2	*VNN3*	0.78	ATF4
							*SESN2*	0.75	ATF4
							*MTHFD2*	0.73	ATF4
							*PYCR1*	0.72	ATF4
							*BACH1*	0.68	Nrf2
**Inhibited genes**	*SLC1A7*	−1.57	ATF4	*TMEM189*	−1.48	ATF4	*COCH*	−1.25	Nrf2
	*PSG5*	−1.43	AhR	*NREP*	−0.99	ATF4	*SNAI2*	−1.20	ATF4
	*PRKAR2B*	−1.23	Nrf2	*KIFC1*	−0.79	ATF4	*INSIG1*	−1.02	Nrf2
	*SOAT2*	−0.80	ATF4	*DLX2*	−0.78	Nrf2	*AKR1B10*	−0.96	Nrf2
	*DAAM2*	−0.78	Nrf2	*BMF*	−0.73	ATF4	*PMAIP1*	−0.88	Nrf2
	*WDR63*	−0.70	AhR	*TGFB2*	−0.72	ATF4	*ANGPTL4*	−0.87	ATF4
	*FAM69A*	−0.68	Nrf2	*DDC*	−0.71	Nrf2	*SNRNP35*	−0.77	ATF4
	*CDH11*	−0.67	Nrf2	*GLI2*	−0.71	ATF4	*SERPINE1*	−0.68	Nrf2
	*LCN2*	−0.66	ATF4	*AURKB*	−0.69	ATF4	*PRC1*	−0.65	Nrf2
	*PLA2G4A*	−0.66	Nrf2	*NEDD9*	−0.67	ATF4	*LMCD1*	−0.64	AhR
	*CXCL5*	−0.64	Nrf2	*TFPI*	−0.65	ATF4	*LBH*	−0.61	Nrf2
	*WISP1*	−0.62	ATF4	*OSMR*	−0.59	Nrf2			
**Activated or Inhibited genes**		**AhR-Nrf2 Overlapping signature**				**Nrf2-ATF4 Overlapping signature**			
		**Genes**	**AhR log**_2_**(FC) averages**	**Nrf2 log**_2_**(FC) averages**		**Genes**	**Nrf2 log**_2_**(FC) averages**		**ATF4 Log2 FC average**
		*NQO1*	0.7	0.83		*ATF3*	0.73		0.90
		*DLGAP5*	−0.64	−0.56		*SLC7A11*	0.70		0.69
		*CFTR*	−0.69	−0.73		*TRIB3*	0.70		1.02
		*RAB39B*	−0.92	−0.52		*CABC1*	0.56		2.90
		*GSTA1*	−1.43	−0.83		*GDF15*	0.48		0.80
						*CCL2*	−0.61		−1.28
						*KCNT2*	−0.9		0.76
**Activated or Inhibited genes**		**AhR-ATF4 Overlapping signature**				**AhR-Nrf2-ATF4 Overlapping signature**			
		**Genes**	**AhR log**_2_**(FC) averages**	**ATF4 log**_2_**(FC) averages**		**Genes**	**AhR Log2 FC average**	**Nrf2 log**_2_**(FC) averages**	**ATF4 log**_2_**(FC) averages**
		*CYP1B1*	3.56	−0.63		*TPX2*	−0.75	−0.8	−2.38

*Gray background indicates genes that appear in the signature of the pathway from previous studies (Supplementary Table 1) and confirmed here. Non-grayed out values are novel allocations from this analysis*.

### Pathways' stratified signatures in liver

#### The three main pathways' stratified signatures in liver

Table 5 shows the stratified signatures in liver of each pathway in four columns (categories): each containing the genes' names and their log2(FC) averages. Genes that appear in more than one column are highlighted in gray and empty lines were left in order to display those genes on the same line in all the categories where they appear. Genes of the first column, sorted by the decreasing absolute values of their log2(FC) averages, appear first, followed by genes appearing in more than one category but not the first column and then the rest of the genes sorted by the decreasing absolute values of their log2(FC) averages as well.

**Table 5:** AhR, Nrf2 and ATF4 pathways' signatures stratified in liver data and by all liver data sub-categories (“Rat Liver *in vitro*” data, “Rat Liver *in vivo*” data and “Human Liver *in vitro*” data).

	**All liver data**		**Rat liver** ***in vitro***		**Rat liver** ***in vivo***		**Human liver** ***in vitro***	
	**Genes**	**Log_2_(FC) averages**	**Genes**	**Log_2_(FC) averages**	**Genes**	**Log_2_(FC) averages**	**Genes**	**Log_2_(FC) averages**
**AhR SIGNATURES**								
Activated genes	*CYP1A1*	4.55	*CYP1A1*	1.30	*CYP1A1*	6.86	*CYP1A1*	4.72
	*CYP1A2*	1.47			*CYP1A2*	1.71	*CYP1A2*	2.44
	*TIPARP*	0.64	*TIPARP*	0.40			*TIPARP*	1.21
			*ABCC4*	0.25	*ABCC4*	0.97	
			*IL1R1*	0.24	*HTATIP2*	1.19	*CYP1B1*	3.49
			*TAF15*	0.22			*SLC20A1*	0.78
Inhibited genes			*PRKAR2B*	−0.20			*KCNT2*	−0.60
			*ANXA1*	−0.18				
			*ANGPTL4*	−0.17				
**Nrf2 SIGNATURES**								
Activated genes	*MAFF*	1.42	*MAFF*	0.67	*MAFF*	2.37		
	*FBXO30*	0.92					*FBXO30*	0.35
	*HSPA1B*	0.82	*HSPA1B*	0.37			*HSPA1B*	0.63
	*PPP1R15A*	0.77			*PPP1R15A*	1.16		
	*GSTP1*	0.67			*GSTP1*	1.24		
	*GCLC*	0.66	*GCLC*	0.35				
	*PSAT1*	0.64			*PSAT1*	1.54		
	*DUSP5*	0.62	*DUSP5*	0.64				
	*SLC3A2*	0.60			*SLC3A2*	1.09	*SLC3A2*	0.40
	*OSGIN1*	0.58			*OSGIN1*	0.91	*OSGIN1*	0.42
	*SLC6A9*	0.57			*SLC6A9*	1.06		
	*SLC20A1*	0.52	*SLC20A1*	0.41				
	*ABCC3*	0.52			*ABCC3*	1.00		
			*YPEL5*	0.47			*YPEL5*	0.37
			*CPT1A*	0.38			*CPT1A*	0.36
	*ASNS*	0.75	*SRXN1*	0.66	*HMOX1*	2.03	*ATF5*	0.37
	*PHGDH*	0.55	*PHLDA1*	0.53	*SLC7A11*	1.74	*AP5Z1*	0.35
	*PLA2G12A*	0.50	*TXNRD1*	0.41	*GDF15*	1.30		
	*SLC7A1*	0.48	*ABCC2*	0.39	*BTG2*	0.89		
			*PIR*	0.34				
			*FLVCR2*	0.33				
			*GSR*	0.33				
			*GABARAPL1*	0.33				
			*AGPAT9*	0.57				
			*TBCEL*	0.48				
			*MMD*	0.33				
Inhibited genes							*MMD*	−0.4
	*LCN2*	−0.45			*LCN2*	−0.97		
			*TGFB2*	−0.34			*TGFB2*	−0.44
	*MID1IP1*	−0.48	*TNFAIP2*	−0.44	*BMF*	−0.88	*ALDH1A1*	−0.61
	*IL33*	−0.46	*VASN*	−0.39	*DHRS7*	−0.69	*DDC*	−0.42
	*NREP*	−0.45	*AURKB*	−0.38			*DUT*	−0.35
	*SERPINB9*	−0.42	*RAB32*	−0.36			*IFIT3*	−0.33
			*CD36*	−0.36			*UGT1A6*	−0.32
			*DCN*	−0.34				
			*CTSC*	−0.34				
			*LBH*	−0.32				
			*CXCL3*	−0.32				
**ATF4 SIGNATURES**								
Activated genes	*TSLP*	1.51					*TSLP*	1.51
	*AKNA*	1.30					*AKNA*	1.30
	*HERPUD1*	1.23	*HERPUD1*	1.28	*HERPUD1*	0.61	*HERPUD1*	2.39
	*IL23A*	1.05	*IL23A*	1.69			*IL23A*	1.86
	*HSPA5*	0.94					*HSPA5*	3.28
	*GTPBP2*	0.91	*GTPBP2*	1.12			*GTPBP2*	1.89
	*PDIA4*	0.87	*PDIA4*	0.92			*PDIA4*	2.18
	*FAM129A*	0.87					*FAM129A*	2.92
	*PYCR1*	0.72	*PYCR1*	0.91				
			*CHAC1*	1.40	*CHAC1*	0.50		
			*KLF15*	0.81	*KLF15*	0.43		
	*SLC1A4*	1.15	*TRIB3*	1.12	*HES1*	0.57	*FIBIN*	2.72
	*NUPR1*	0.94	*BCAT2*	0.97	*USP2*	0.55	*LCN2*	1.91
	*LONP1*	0.80	*ARHGEF2*	0.93	*ENC1*	0.48	*CTH*	1.62
	*VNN3*	0.78	*CASP4*	0.84	*TSC22D3*	0.44	*NFE2L1*	1.2
	*SESN2*	0.75	*KLF4*	0.82	*DDIT4*	0.39		
	*BACH1*	0.68	*BET1*	0.82	*SLC38A2*	0.38		
			*WARS*	0.80	*IP6K2*	0.62		
			*PCK2*	0.73				
			*SLC25A33*	0.71				
			*SLC7A5*	0.71				
			*ACOT2*	0.83				
			*MANEA*	0.75				
Inhibited genes	*PRC1*	−0.65	*PRC1*	−0.61				
	*LMCD1*	−0.64	*LMCD1*	−0.80			*LMCD1*	−1.73
	*LBH*	−0.61					*LBH*	−2.56
	*SNAI2*	−1.20	*DPYSL2*	−0.98	*FOXA2*	−0.61	*FRMD6*	−1.52
	*AKR1B10*	−0.96	*DUSP6*	−0.97	*ABCG2*	−0.49	*SLC39A10*	−1.35
	*PMAIP1*	−0.88	*IFIT3*	−0.72	*NEDD9*	−0.43	*GPNMB*	−1.26
	*SNRNP35*	−0.77	*EMILIN1*	−0.69	*TMEM159*	−0.37	*ANKRD1*	−1.16
	*SERPINE1*	−0.68	*FCER1G*	−0.65			*PHLDA1*	−1.16
			*SQRDL*	−0.61				
			*IFI44*	−0.61				

*Genes that appear in more than one column are highlighted in gray*.

##### AhR stratified signatures

Table 5 shows that *CYP1A1* is clearly, by far the most activated gene in this pathway. Three other genes appear in the AhR signature in more than one column: *CYP1A2* everywhere except “Rat liver *in vitro*,” *TIPARP* everywhere except “Rat liver *in vivo*” and *ABCC4* shows up in these two categories only. “Rat liver *in vitro*” AhR signature is completed by five additional genes, “Rat liver *in vivo*” by one more and “Human liver *in vitro*” by three.

##### Nrf2 stratified signatures

Nrf2 signatures are bigger: 22 genes in the all liver data signature, 28 for “Rat Liver *in vitro*” and 15 for each of “Rat Liver *in vivo*” and “Human Liver *in vitro*”. Around two third of those genes are “Activated genes” and the rest have negative log2(FC) averages. *MAFF, SLC3A2, OSGIN2* are among the “Activated genes” that appear in three out of the four categories we are studying. Other important genes show up in two columns (*HSPA1B, PPP1R15A*, and *GCLC*) and some, in only one (*SRXN1* in “Rat Liver *in vitro*” and *HMOX1* in “Rat Liver *in vivo*”). The values of the “Rat liver *in vivo*” are also higher than the “Rat liver *in vitro*” and “Human liver *in vitro*” categories.

##### ATF4 stratified signatures

ATF4 signatures size is similar to Nrf2's signatures with a comparable proportion of activated genes: 23 genes in the all liver data signature, 28 for “Rat liver *in vitro*” and 14 for each of “Rat liver *in vivo*” and 19 for “Human liver *in vitro*.” *HERPUD1* is an important gene in this pathway; it is part of the signature of every single category we are examining and exhibits values as high as 2.39 in “Human Liver *in vitro*” (among the highest in ATF4 signatures). Other genes also are present in the majority of the categories: *IL23A, GTPBP2*, and *PDIA4*. It is noteworthy that the ATF4 signature of “Rat Liver *in vivo*” results don't have a lot in common with the other three categories and its log2(FC) averages are lower than the rest (the highest value is 0.61 for *HERPUD1*).

#### The overlapping zones stratified signatures

**Figure 5:**
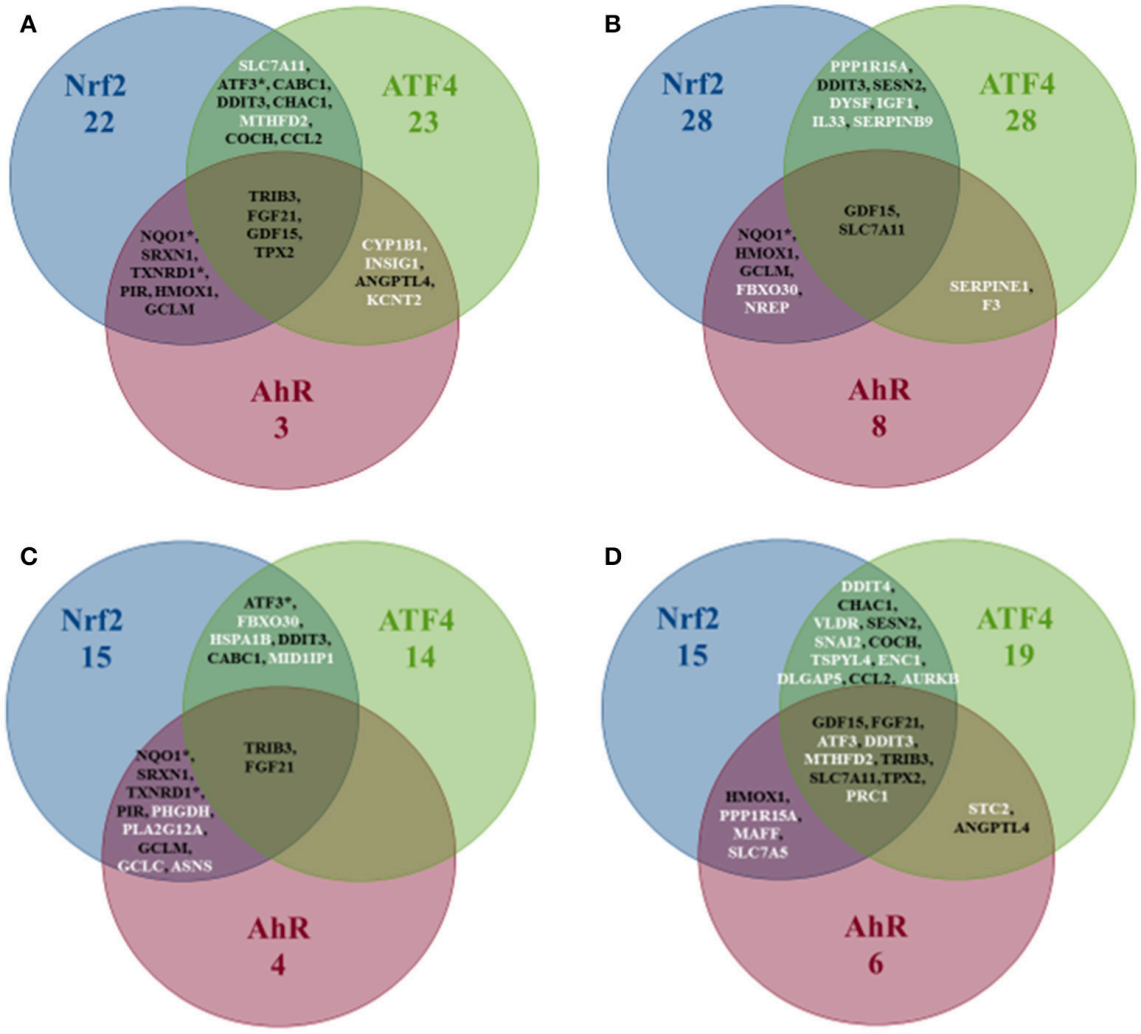
Venn diagram of the number of genes per pathway's stratified signatures and names of genes of overlapping zones. Categories: **(A)** All liver data, **(B)** Rat Liver *in vitro* data, **(C)** Rat Liver *in vivo* data, **(D)** Human Liver *in vitro* data. *Refers to genes that were known to be part of the same overlapping zone according to Supplement Table 1 lists. White is the color of gene names that appear in an overlapping zone of only one of the four categories studied, and black is the color of gene names that appear in more than one category (two, three or four).

Figure 5 shows that the AhR-ATF4 overlapping zone is the least populated (four genes maximum in all liver data, no genes for “Rat Liver *in vivo*” and two genes in the two other categories). The number of genes in the AhR-Nrf2 overlapping signatures ranges from four to eight, with many typical key Nrf2 genes (*NQO1, SRXN1, HMOX1, TXNRD1*, and *GCLM*) appearing in more than one category. The Nrf2-ATF4 overlapping signatures contain six to eleven genes (*DDIT3, ATF3*, and *CHAC1* are among the repetitive genes). Finally, *TRIB3, FGF21, GDF15, SLC7A11*, and *TPX2* are in the signature of the zone mutual to all three pathways for at least two of the four categories studied.

### Pathways' stratified signatures in liver

**Figure 6:**
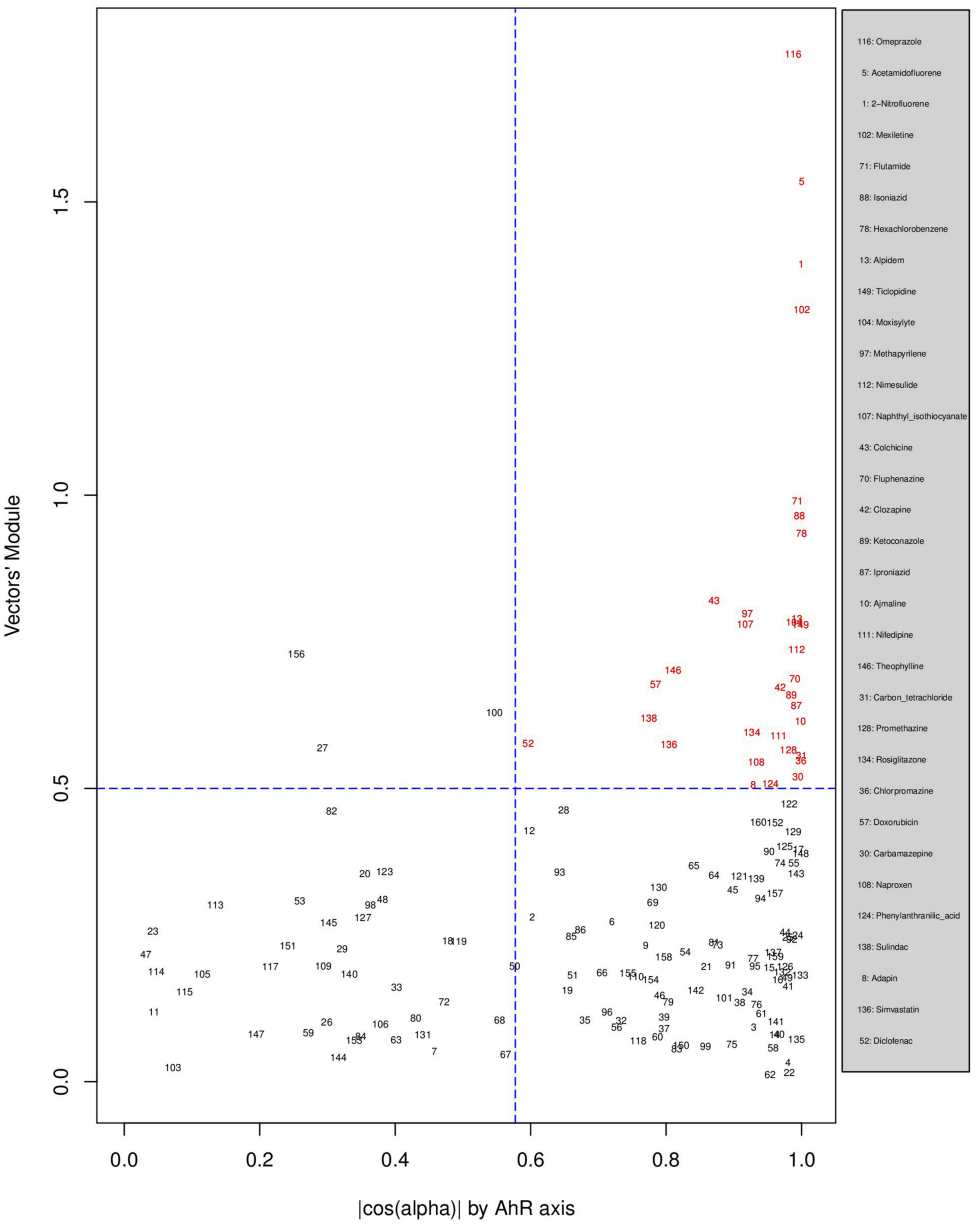
Distribution of chemicals by potency (Y-axis: module $\left||\vec{OK}|\right|$ of the vector linking the origin O(0,0) to the chemical's point in a 3D space) and specificity to the AhR pathway (X-axis: the absolute value of the **| cos(α)|** of the angle between $\vec{OK}$ and the AhR axis in a 3D space). Chemicals are represented by their rank in the alphabetically ordered list. Chemicals that are both strong (horizontal blue dashed line: $\left||\vec{OK}|\right|>0.5)$ and AhR specific (vertical blue dashed line: $cos(\alpha )\;=\frac{1}{\sqrt{3}}\;$) are in red and their names are listed in the legend on the right.

**Figure 7:**
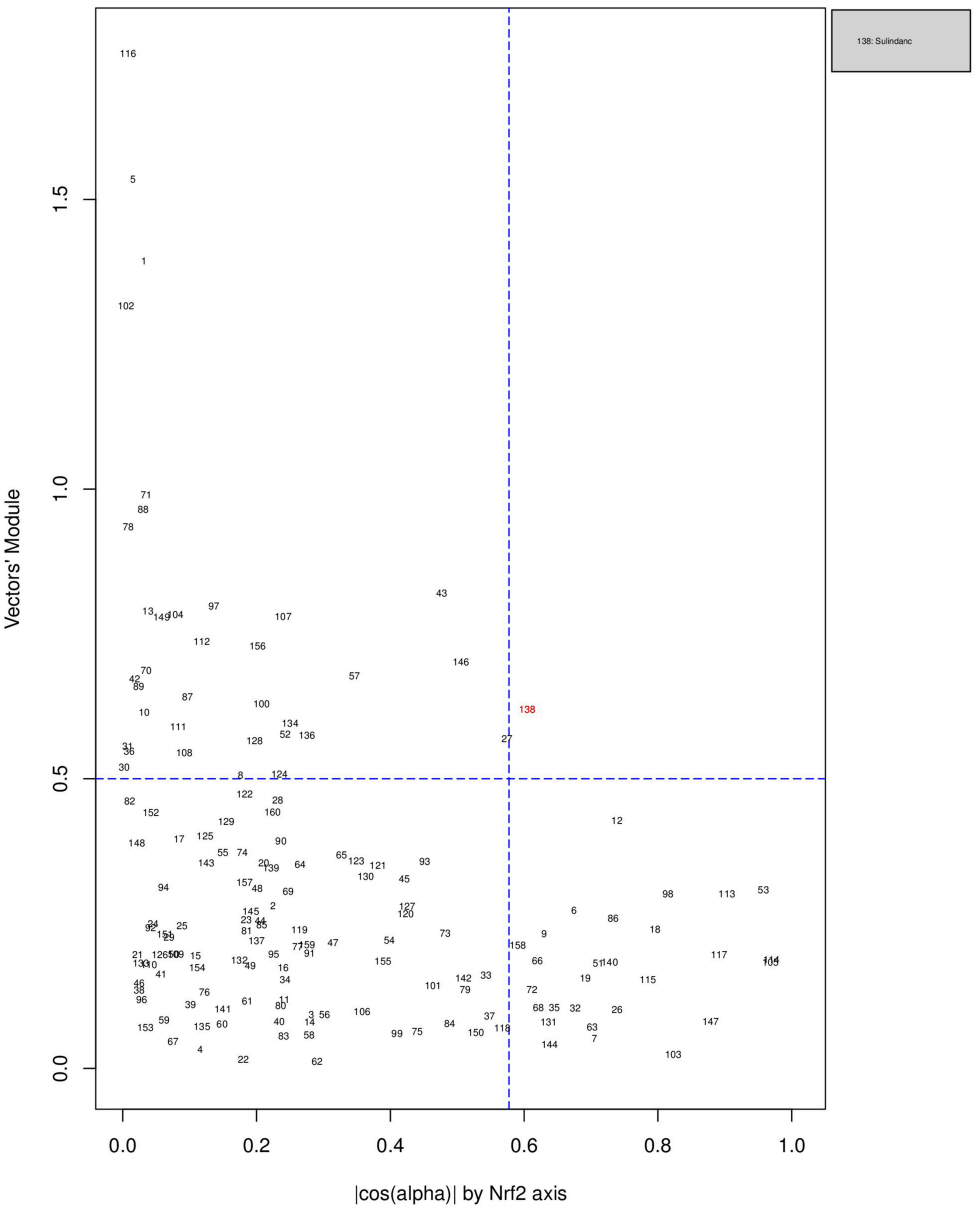
Distribution of chemicals by potency (Y-axis: module $\left||\vec{OK}|\right|$ of the vector linking the origin O(0,0) to the chemical's point in a 3D space) and specificity to the Nrf2 pathway (X-axis: the absolute value of the **|cos(α)|** of the angle between $\vec{OK}$ and the AhR axis in a 3D space). Chemicals are represented by their rank in the alphabetically ordered list. The only chemical that is both strong (horizontal blue dashed line: $\left||\vec{OK}|\right|>0.5)$ and AhR specific (vertical blue dashed line: $cos(\alpha )\;=\frac{1}{\sqrt{3}}\;$) Sulindac, is in red and it is listed in the legend on the right.

**Figure 8:**
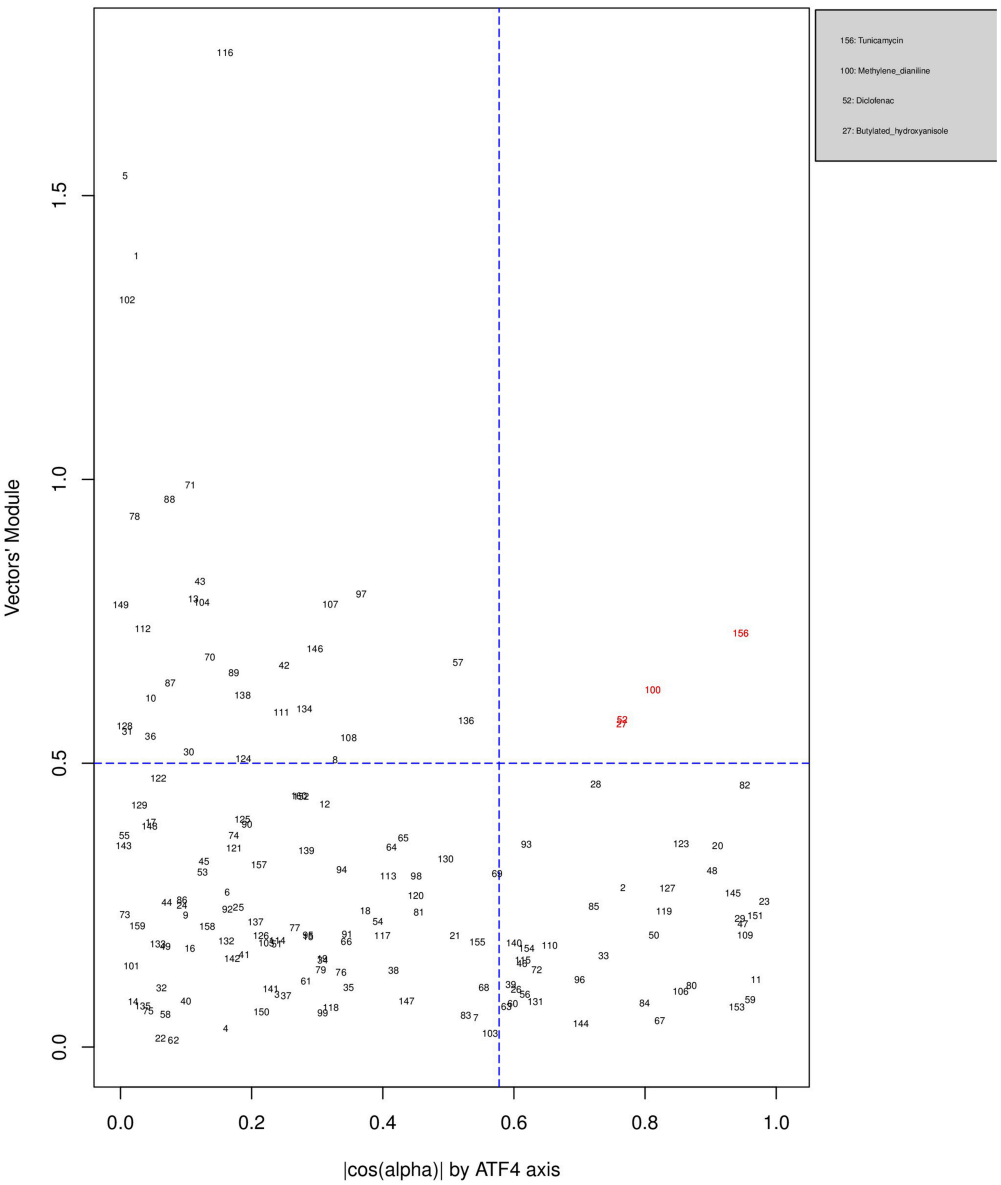
Distribution of chemicals by potency (Y-axis: module $\left||\vec{OK}|\right|$ of the vector linking the origin O(0,0) to the chemical's point in a 3D space) and specificity to the ATF4 pathway (X-axis: the absolute value of the **|cos(α)|** of the angle between $\vec{OK}$ and the AhR axis in a 3D space). Chemicals are represented by their rank in the alphabetically ordered list. Chemicals that are both strong (horizontal blue dashed line: $\left||\vec{OK}|\right|>0.5)$ and AhR specific (vertical blue dashed line: $cos(\alpha )\;=\frac{1}{\sqrt{3}}\;$) are in red and their names are listed in the legend on the right.

Figures 6, 7, 8 plot the 160 chemicals' vector modules vs. the absolute value of **cos** **(α)**, which represents the pathway activation scores of chemicals that activate each pathway both selectively and strongly. Chemicals are represented by a number that corresponds to their rank in the alphabetically ordered list. The blue dashed lines mark the vertical $\left(cos(\alpha )\;=\frac{1}{\sqrt{3}}\;\right)$ and horizontal ($\left||\vec{OK}|\right|=0.5$) limits we set. The number chemicals that are off these limits is 34 for AhR, one for Nrf2 and four for ATF4; these chemicals are in red and their names are listed in the legend on the right by the order of the decreased values of the product result $cos(\alpha )\times \left||\vec{OK}|\right|$. As we can see in these figures' legends, “pathway specific activators” show up first in the lists of AhR (Omeprazole) and ATF4 (Tunicamycin), but do not appear at all in the list of Nrf2 (Phorone).

## Discussion

Nrf2, ATF4, and AhR are important TFs in toxicological contexts and have well described downstream gene targets (Jennings et al., 2013). Each of these TFs have distinct unrelated upstream activation points, unique gene targets, but also have direct (i.e., via multiple upstream promoter regions) and likely indirect overlaps on some specific gene targets. The AhR protein is a cytosolic protein receptor, where activation via chemical ligand binding causes nuclear translocation, DNA binding to it consensus sequence and RNA transcription. Several toxic compounds including dioxin-like compounds activate AhR. The TF Nrf2 is liberated from its cytosolic inhibitor KEAP1, where the latter is sensitive to electrophiles and ROS. The TF ATF4 is activated via PERK, where PERK is activated when its inhibitor BiP, dissociates from PERK to bind unfolded proteins. All sorts of Endoplasmic Reticulum disturbances can cause an increase in unfolded proteins.

Using multiple toxicogenomic databases we investigated the most appropriate activators of these three pathways, where it is expected that the chemical does not directly activate the other two pathways. These compounds were, Benzo(a)pyrene and Omeprazole for AhR, Potassium Bromate and Phorone for Nrf2 and Tunicamycin A for ATF4. All conditions up to and including 24 h were pooled to generate a list of genes allocated to the three pathways (Table 4). This list confirmed the majority of a *priori* literature based information of “Activated genes” (i.e., upregulated). Although some genes were now reallocated to different pathways. The overlap with “Inhibited genes” (i.e., down regulated), was much poorer. This is too be expected as TF activated gene down regulation is much more complex and is often due to competition for auxiliary transcription facilitating proteins. Cytochrome P450 1A was the central element of the AhR pathway: *CYP1A1* is the most prominent gene of this pathway, regardless of the experimental category, followed by *CYP1A2*. These findings are similar to previous investigations and have been implemented in a systems biology model (Hamon et al., 2014). For the Nrf2 pathway, the prototypical Nrf2 genes (*HMOX1, SRXN1*, and *GCLM*) appear in the Nrf2 signature of all datasets, but also in the AhR-Nrf2 overlapping signature for most liver categories. This may reflect the fact that several AhR agonists are themselves metabolized to reactive chemicals via AhR dependent CYP expression. For example benzo(a)pyrene is a substrate of the CYP1 sub family of cytochrome P450 enzymes, and it promotes its own metabolism to reactive epoxide and quinone products (Gelboin, 1980). These metabolic products can lead to oxidative stress and to an activation of the Nrf2 pathway as part of a second line of responses (Burchiel and Luster, 2001). The only activated gene that appears in the ATF4 signature of each of the three studied categories is HERPUD1. In most cases, HERPUD1 also had the highest log2(FC) averages. Overlapping zones show an interaction between AhR and Nrf2, between Nrf2 and ATF4, but a very limited or non-existent interaction between AhR and ATF4 pathways.

We have used the exclusive pathway genes to create pathway chemical activation capacity (CAC) scores. The CAC reflects both specificity for the pathway (**cos** **(α)**) and the activation potency $\left||\vec{OK}|\right|$. CAC scores were generated for 160 chemicals using the TG-GATEs liver data. For ATF4, tunicamycin, methylene dianiline, diclofenac, and butylated hydroxyanisole were ranked highest, in that order. Tunicamycin was used as a specific ATF4 specific activator. Both diclofenac and butylated hydroxyanisole have previously been demonstrated to positive modulate the ATF4 pathway (Afonyushkin et al., 2010; Fredriksson et al., 2014). The molecular mechanism for methylene dianiline has not been fully elucidated and this evidence would suggest an ER disturbance and/or proteotoxic mechanism. For AhR, 34 chemicals were considered positive by CAC scores. Omeprazole was ranked highest, followed by acetamidofluorene, 2-Nitrofluorene, mexiletine, flutamide, isoniazid, and hexachlorobenzene. Many of the 34 chemicals have not been previously linked with AhR, but several are. These include, hexachlorobenzene (Randi et al., 2008; de Tomaso Portaz et al., 2015), ketoconazole (Novotna et al., 2014), clozapine (Donohoe et al., 2008), and doxorubicin (Volkova et al., 2011). Fluphenazine has not been established as a ligand for the AhR, its structure—a halogenated aromatic ring system—closely matches the motif involved in binding to this receptor (Donohoe et al., 2008). In a recent study we have demonstrated that isoniazid induced CYP1A1 in HepaRG cells, which is a potential indicator of AhR activation (Limonciel et al., 2018). Only Sulindac from the 160 was ranked as active using the CAC selection criteria, which may seem surprising given the frequency of oxidative injury in liver toxicities. Although butylated hydroxyanisole was marginal. The reason for a lack of Nrf2 activation prediction might be simply due to the fact that none of the 160 compounds, including the positive compound phorone cause an Nrf2 response in the liver within the first 24 h. Another possibility is that removing the overlapping genes has weakened the ability to pick up this pathway. Indeed, this is a weakness in the overall strategy as it is difficult to determine in such data sets if the pathways themselves are co-regulated since there are several gene overlaps amongst the pathways.

## Summary and conclusion

The size of the data set, its multiple sources, abundancy of compounds, concentrations and time of exposures, *in vitro* and *in vivo*, different organs are both a blessing and a curse. On the one hand, it is generally an advantage to have as broad as data set as possible, but the different sizes and focuses of the individual data sets/studies meant we needed to reduce the data to the lowest denomination. Another major issue was the low abundance of well described pathway activators. Despite these issues we have made some interesting observations and have developed a method to quantify a chemical's capacity to activate one three pathways.

We uncovered variations in AhR, ATF4 and Nrf2 signatures across tissues, compounds, species and *in vivo* vs. *in vitro*. Some of these alterations are likely to be linked to pharmacokinetics, including distribution and metabolism, others may be linked to tissue specific regulation of these pathways. While some genes were very variable across experimental conditions, some were extremely robust, for example CYP1A1 in the AhR pathway and HERPUD1 in the ATF4 pathway. Some genes swing between a pathway's specific signature and overlapping zones for example *GCLC* between Nrf2 and AhR-Nrf2. Others are regularly on overlapping signatures for example *TPX2* and *TRIB3*. However, it is not possible with this type of analysis to delineate whether these overlaps are solely on a gene level or also on the pathway level.

The CAC score system developed, based on $\cos\left(\alpha \right)\times ||\vec{OK}||$, can be used to quantify a chemical's specificity and potency to selectively activate one of these pathways. However, future work will be required to validate and optimize the gene signatures utilized.

## Data availability statement

The dataset used for this analysis can be found in an Excel document in the Supplementary Material.

## Author contributions

FB and PJ conceived of the original idea and supervised the findings of this work. EZ, FB, PJ, AL, and AW planned the simulations. FB and PJ developed the theoretical formalism and verified the analytical methods. Supervised by PJ and FB, EZ performed the analytic computations and the numerical simulations. SW and BvdW generated target gene lists. XJ and AK-S designed the figures. EZ wrote the manuscript with support from FB, PJ, and AL and then all authors reviewed and commented the final manuscript. All authors provided critical feedback and helped shape the research, analysis and manuscript.

### Conflict of interest statement

The authors declare that the research was conducted in the absence of any commercial or financial relationships that could be construed as a potential conflict of interest.

## Footnotes

^1^http://wwwdev.ebi.ac.uk/fg/dixa/

^2^https://dbarchive.biosciencedbc.jp/en/open-tggates/desc.html

^3^http://nmg-r.bioinformatics.nl/NuGO_R.html

^4^https://biodbnet-abcc.ncifcrf.gov/

## Abbreviations

AhR: Aryl Hydrocarbon Receptor
ATF4: Activating Transcription Factor 4
CAC: Chemical Activation Capacity
ChIP-seq: Chromatin Immunoprecipitation followed by DNA sequencing
CYP1A1: Cytochrome P1-450 A1
FC: Fold Change
Nrf2: Nuclear Factor (Erythroid-derived 2)-Like 2 (NFE2L2)
TF: Transcription Factors.
