# Corrigendum: Investigation of Nrf2, AhR and ATF4 Activation in Toxicogenomic Databases

**DOI:** 10.3389/fgene.2019.00517

**Published:** 2019-06-04

**Authors:** Elias Zgheib, Alice Limonciel, Xiaoqi Jiang, Anja Wilmes, Steven Wink, Bob van de Water, Annette Kopp-Schneider, Frederic Y. Bois, Paul Jennings

**Affiliations:** ^1^Laboratoire de Biomécanique et Bio-ingénierie, Sorbonne Universités – Université de Technologie de Compiègne, Compiègne, France; ^2^Division of Molecular and Computational Toxicology, Amsterdam Institute for Molecules, Medicines and Systems, Vrije Universiteit Amsterdam, Amsterdam, Netherlands; ^3^Division of Biostatistics, German Cancer Research Center, Heidelberg, Germany; ^4^Division of Drug Discovery and Safety, Leiden Cell Observatory High Content Imaging Screening Facility, Leiden Academic Center for Drug Research, Leiden University, Leiden, Netherlands; ^5^Models for Ecotoxicology and Toxicology Unit (DRC/VIVA/METO), Institut National de l'Environnement Industriel et des Risques, Verneuil-en-Halatte, France

**Keywords:** transcriptomics, Nrf2, AhR, ATF4, toxicity pathways, toxicogenomic, oxidative stress

In the original article, we neglected to mention that this work was partly supported by the EU-ToxRisk project (An Integrated European “Flagship” Program Driving Mechanism-Based Toxicity Testing and Risk Assessment for the 21st Century) funded by the European Commission under the Horizon 2020 programme (Grant Agreement No. 681002).

A correction has therefore been made to the **Acknowledgments**:

“The research leading to these results has received support from the Innovative Medicines Initiative Joint Undertaking (IMIJU) under grant agreement number 115439, resources of which are composed of financial contribution from the European Union's Seventh Framework Programme (FP7/2007-2013) and EFPIA companies' in kind contribution. This work was supported by the 2015 CEFIC-LRI award (AL) and partly supported by the EU-ToxRisk project (An Integrated European “Flagship” Program Driving Mechanism-Based Toxicity Testing and Risk Assessment for the 21st Century) funded by the European Commission under the Horizon 2020 programme (Grant Agreement No. 681002). This publication reflects only the author's views and neither the IMI JU nor EFPIA nor the European Commission are liable for any use that may be made of the information contained therein.”

The authors apologize for this error and state that this does not change the scientific conclusions of the article in any way. The original article has been updated.

