# Supplementary material for: Investigation of Nrf2, AhR and ATF4 Activation in Toxicogenomic Databases
Source: Front Genet. 2018 Oct 2;9:429. doi: 10.3389/fgene.2018.00429 (PMC6176024; doi:10.3389/fgene.2018.00429)
Supplement: Supplementary file 4 [file Data_Sheet_1.pdf]

Supplementary Material

Investigation of Nrf2, AhR and ATF4 activation in toxicogenomic databases

Elias Zgheib*, Alice Limonciel, Xiaoqi Jiang, Anja Wilmes, Steven Wink, Bob van de Water, Annette Kopp-Schneider, Frederic Y. Bois*, Paul Jennings

*** Correspondence:**
Frederic Y. Bois
frederic.bois@ineris.fr

Elias Zgheib
elias.zgheib.pro@gmail.com

1 Supplementary Tables

Supplementary Table 1. Target gene lists for AhR, Nrf2 and ATF4.

**Pathway** (Number of genes)	**Genes** (804)											
**AhR** (60)	AHRR	ALDH1A3	ALS2CL	ATXN1	BATF	BCL3	BMF	CACNA1D	CYP1A1	CYP1A2	CYP1B1	DDX24
	DHX37	DLL1	DLX1	DNMBP	DPP9	EDC3	ELF4	EREG	FAM32A	FAM65C	FLVCR2	FOSL2
	FREM2	IGF1R	LAMA3	LMCD1	MAPRE2	NEDD9	NIN	NPY1R	PITPNM2	PLEC	PRPS1	PSG5
	PYGL	RND1	RRP12	RUNX1	RUNX2	SAMD12	SAT1	SECTM1	SIPA1L2	SLC27A2	SLC2A11	STRBP
	TFAP2A	TH	TIPARP	TMEM45B	TPCN1	TRAFD1	TRUB2	USP3	VDR	VIPR1	VTCN1	WDR63
**Nrf2** (306)	ABCB6	ABCC1	ABCC2	ABCC3	ABCC5	ABHD4	ACAP2	ADAM23	ADCY1	ADO	AGPAT9	AHR
	AIFM2	AKAP7	AKR1B10	AKR1C1	AKR1C2	AKR1C3	ALDH1A1	ALS2	AMBP	ANKRD44	ANXA1	AOX1
	AP5Z1	APPL2	ARHGAP18	ARHGDIB	ASB3	ATF4	ATG16L2	AURKB	B3GNT2	BACH1	BBS9	BCL2
	BEND6	BET1	BLVRB	BMP8A	BMPER	BMPR1A	BTG2	C12orf29	C16orf72	C8orf4	C9orf3	CABC1
	CAPN7	CBARA1	CBR3	CCDC104	CCDC109B	CCDC53	CCDC90B	CD27	CD302	CD36	CDC2L6	CDH11
	CDKN2B	CES1	CFTR	CHPT1	CLCN1	CLIP4	CLTC	COCH	COL24A1	COL4A1	COLEC12	
		COMMD6										
	CORO7	COX6C	CPNE8	CPT1A	CTSB	CTSC	CTSO	CTTN	CXCL5	DAAM2	DAGLB	DCN
	DENND4C	DHX40	DLGAP5	DUSP22	DUSP5	ECM1	EDA2R	EMC7	ENC1	ENTPD5	EPHX1	EXOC7
	FAM102A	FAM167A	FAM210B	FAM214A	FAM69A	FBF1	FBXL3	FBXO30	FECH	FKBPL	FNBP1	FOPNL
	FOXA2	FRMD6	FTH1	FUK	FZD6	GABARAPL1	GAS1	GCLC	GCLM	GCNT3	GDF15	GDI2
	GLI2	GLO1	GPNMB	GRIP2	GRM1	GSR	GSTA1	GSTA4	GSTM1	GSTM2	GSTM3	GSTP1
	GVIN1	HACE1	HBP1	HECA	HES1	HINT3	HIST1H1C	HMHB1	HRASLS2	HRSP12	HSPA1B	IDH1
	IDS	IFIT3	IGF1	IGF2R	IL33	IL6	INSIG1	INSIG2	IRF4	ITGB2	JAG1	
		KCNAB1										
	KDELR2	KEAP1	KIF26B	KLHDC8A	KRAS	LAPTM4B	LAYN	LBH	LMF1	LNX1	LPL	
		LRRC4C										
	LTA	LYPD6B	LYPLAL1	MACF1	MAFF	MAFG	MAGOHB	MAMDC2	MAP3K9	MAPK8	MATN2	ME1
	MEGF9	MEIS1	MFSD9	MGST2	MLYCD	MMD	MRPL14	MRPL33	MSC	MT2A	NCAPD2	NETO2
	NPNT	NQO2	NREP	OAT	OIT3	OSGIN1	P2RY10	PAIP1	PBX1	PCDH7	PDDC1	PDGFC
	PDIA4	PGAP1	PHLDA1	PION	PIP5K1C	PIR	PLA2G4A	PLN	PMAIP1	PPP1R12B	PQLC3	PRC1
	PRDM1	PRDX1	PRKAR2B	PRKCD	PRR13	PSMB5	PTCD1	PTGR1	PTTG1IP	RAB31	RAB32	
		RAB33A										
	RFFL	RGS10	RND3	RNF121	RNF141	RNF216	RPL10A	RPS6	RXRA	SARM1	SCAMP1	SCYL1
	SDCCAG8	SDPR	SEC63	SEMA3E	SERPINE1	SH3RF2	SH3TC1	SIM2	SLC12A8	SLC16A6	SLC16A9	
	SLC39A10											
	SLC44A3	SLMAP	SNX1	SNX13	SNX22	SNX4	SOAT1	SOCS5	SOD1	SOD2	SQRDL	SRP9
	SRXN1	SSR3	SUB1	SYNPO2	TALDO1	TBC1D23	TBCEL	TBXAS1	TFE3	TFPI	TGFB2	TINAG
	TKT	TMEFF2	TMEM159	TMEM206	TMEM63A	TMEM64	TNFSF9	TRIM69	TSPAN3	TXLNB	TXN	TXNDC5
	UBE2V2	UGT1A1	UGT1A6	UGT2B7	UNKL	VASN	VPS8	VSTM4	WAPAL	WASF1	WDR81	WDSUB1
	WNT5A	YAF2	ZC3H11A	ZDHHC20	ZFAND2B	ZMAT4						
**ATF4** (408)	AARS	ACACA	ACAD8	ACOT2	ACOX2	ADM2	AFF1	AGAP1	AKAP2	AKNA	ALDH18A1	
		ALDH1L2										
	ALKBH5	AMACR	ANAPC1	ANGPTL4	ANGPTL6	ANK2	ANKRD1	ANKRD11	ANP32B	APBB2	APOBEC1	APOE
	ARHGEF2	ARID5B	ARPC5L	ASCC2	ASNS	ATF5	ATF6	ATL2	ATP6V1G1	ATXN2L	B4GALNT2	B9D2

	BCAR1	BCAT1	BCAT2	BCMO1	BDNF	BGLAP	BHLHE22	BNIP1	BOLA1	BTF3L4	CA9	CABP1
	CALCRL	CAMSAP1	CARS	CASC5	CASP4	CAST	CCL2	CCL7	CCT8L1	CD276	CD74	CDH24
	CDSN	CEBPB	CEBPG	CHAC1	CLCN3	CLIC4	CNOT1	CNPY2	COASY	COL18A1	CP	CRLS1
	CRYBG3	CTH	CUL2	CXADR	CXCL3	CXCR5	CYB5R1	DAAM1	DDIT3	DDR2	DEGS2	DHRS7
	DLEU7	DNAJA3	DNAJB12	DPF2	DPYSL2	DTNBP1	DUSP6	DUT	DYSF	ECHS1	EDEM3	EIF1
	EIF2S2	EIF3C	EIF4EBP1	EIF4G2	EIF5	ELMSAN1	EMILIN1	EPO	EPRS	ERLIN1	ERO1L	ETF1
	ETS1	F3	FADS3	FAM119A	FAM129A	FAM159A	FAM175A	FAM175B	FAM188B	FAM26F	FAM83F	
		FAM96A										
	FBXL2	FBXO11	FBXO31	FCER1G	FGF19	FGF21	FIBIN	FLAD1	FLNC	FLRT1	FNDC7	GARS
	GHITM	GNG5	GNPNAT1	GOT1	GPAM	GPATCH3	GPR85	GPT2	GPX4	GRB10	GSK3A	GSTO1
	GTPBP2	GTPBP4	H2AFZ	HAS2	HAX1	HDAC8	HERPUD1	HFE	HHIPL1	HSPA5	HTRA1	IARS
	ICK	IER2	IFI44	IFT172	IHH	IL1R1	IL2	IL23A	INCENP	INPP4B	INPP5B	IP6K2
	IPMK	ITGB5	ITGB7	ITIH1	JAGN1	JDP2	JHDM1D	KBTBD5	KCNT2	KDM5A	KDM6B	
		KHNYN										
	KIF13B	KIFC1	KLF15	KLF4	KLHDC10	KRTCAP2	LARS	LCN2	LEPREL1	LEPROTL1	LGALS3	
		LGALS8										
	LHFPL2	LMBRD1	LMO4	LONP1	LRRK1	LSG1	LSM14A	LTBP3	MACROD1	MANEA	MARS	MAT2B
	MAZ	MCL1	METTL9	MFF	MID1IP1	MMP19	MRAS	MRPL13	MRPL24	MRPL54	MTHFD1L	
		MTHFD2										
	MTHFR	MTM1	MYCBP2	MYOM1	NAAA	NARS	NCL	NDRG1	NDUFA4L2	NF1	NFE2L1	NFKB1
	NFU1	NIPBL	NME2	NOS2	NOSIP	NRBF2	NRIP2	NUPR1	OPTC	ORMDL3	OSMR	OTUB2
	PACSIN2	PAQR3	PAX8	PCK2	PDAP1	PDE1A	PDE4DIP	PER2	PER3	PFDN1	PHF10	
		PHF21A										
	PHGDH	PHYHD1	PIK3C2G	PKMYT1	PLA2G12A	PLD1	PLEKHH3	PNRC2	POP5	PPP1R15A	PPP1R15B	
		PPP2R5A										
	PRDM15	PRKG2	PRSS35	PRSS36	PSEN1	PTBP1	PTGS2	PTN	PTPN12	PTPN21	PTPRS	PVRL2
	PYCR1	RAB39B	RAD21	RAI1	RAN	RBM39	RBM4	RBM9	RCC2	RD3	RELN	
		RHBDD1										
	RHOQ	RNF114	RPL13A	RPL7	RPRD2	RPS6KA2	RSBN1	RUNX3	RUVBL2	SAMD8	SARS	
	SERPINB9											
	SERPINF1	SERTAD2	SESN2	SH3BP5L	SHMT2	SLC16A14	SLC19A2	SLC1A4	SLC1A5	SLC1A7	SLC20A1	
	SLC25A28											
	SLC25A33	SLC25A39	SLC35B4	SLC38A2	SLC38A7	SLC41A3	SLC6A9	SLC7A1	SLC7A11	SLC9A9	SLIT1	SLTM
	SNAI2	SNRNP35	SNX12	SNX33	SOAT2	SPATS2	SPEN	SRPK1	SRSF1	STARD5	STC2	
		STEAP1										
	STK40	SURF6	SYNJ2	TAB2	TAF15	TAF5	TAGLN2	TARS	TBC1D9B	TBPL1	TET3	TGIF1
	TLCD2	TMEM11	TMEM154	TMEM189	TMEM74	TMIGD1	TNFAIP2	TNFAIP6	TNKS	TNPO1	TNRC6C	TOR3A
	TPX2	TRAF3IP2	TRIB3	TRIM35	TRIM66	TSC22D3	TSLP	TSPAN5	TSPYL4	TTC23	TTC9C	UBA3
	UBE2L3	UBE2W	UBP1	UBR2	UBR4	UHRF1BP1	UQCRC2	UQCRQ	USP11	USP2	USP36	
		USP6NL										
	VAC14	VARS	VLDLR	VNN3	VPS54	WARS	WBP4	WDR27	WISP1	WWP2	WWTR1	XPOT
	YARS	YLPM1	YWHAG	ZAK	ZBTB38	ZBTB7B	ZBTB7C	ZC3H18	ZC3HAV1	ZFC3H1	ZNF268	
		ZYG11B										
**AhR Nrf2** (7)	ABCG2	DLX2	MCOLN2	NFE2L2	NQO1	PSPC1	TXNRD1					
**AhR ATF4** (4)	CRISPLD2	DDIT4	SLC7A5	UBE2G2								
**Nrf2 ATF4** (17)	ABCC4	ATF3	CLN5	CR1L	DDC	DHX57	GOLIM4	HMOX1	HTATIP2	MDFIC	PSAT1	SCPEP1
	SEMA6D	SPRED1	SQSTM1	TBCE	YPEL5							
**AhR Nrf2 ATF4** (2)	SLC3A2	TMTC2										
**Genes with no data*** (53)	**Nrf2** (16) : AI646023, AKR1A4, BC067068, BCLX, CYP2A5, GSPDX, H2-T10, IQWD1, NDP52, PREI4, PRX1, SNF1LK2, SPEER3, SPEER4A, TXN1, ZFP51.<br>**ATF4** (36) : ABCB1A, ACAA1A, B230217C12RIK, BB014433, BC019943, BC055111, CYP2AB1, D48WG0951E, DEB1, DEFB43, DNAIC2, GCFC1, GM129, GM5627, GM5820, GM5867, H2-Q7, HIST2H4, IFITM6, JMJD3, LIPIN2, MTAP4, PGPEP1L, RNASET2B, SAMD4, SERPINA3C, SLCO1A5, TREM3, UGT1A6A, VEGF, ZFP238, ZFP365, ZFP598, ZFP608, ZFP623, ZFP708.<br>**Nrf2 ATF4** (1) : B230315N10RIK.<br><br>***** No data are available for these genes in the carcinoGENOMICS, PREDICT-IV, or TG-GATEs databases.											

Supplementary Table 2. Chemicals used in each experimental category.

**Carcino-GENOMICS**	Human	Kidney	*in vitro*	Bolus	(30)	1-Amino-2,4-dibromoanthraquinone, 2-Nitrofluorene, 4-Acetylaminofluorene, Aristolochic acid, Benzo(a)pyrene, Benzoin, Benzyl alcohol, Bromodichloromethane, Chlorothalonil, Citrinin, Clonidine, Cyclosporine A, D-mannitol, DCVC, Diclofenac, DL-Menthol, Fumonisin-B1, Hydroquinone, Lead-(II)-acetate-trihydrate, Monuron, N-Ethyl-N-(2-hydroxyethyl)nitrosamine, N-Nitrosomorpholine, Nifedipine, Nitrilotriacetic acid, Ochratoxin A, Potassium bromate, Streptozotocin, Tolbutamide, Triclosan (Igarsen), Tris(2,3-dibromopropyl)phosphate.
	Rat	Kidney	*in vitro*	Bolus	(15)	2-Nitrofluorene, Aristolochic acid, Benzo(a)pyrene, Bromodichloromethane, Chlorothalonil, Clonidine, D-Mannitol, DCVC, Diclofenac, Dimethylnitrosamine, Monuron, Nifedipine, Ochratoxin A, Potassium bromate, Tolbutamide.
**PREDICT-IV**	Human	Kidney	*in vitro*	Repeat	(12)	Adefovir dipivoxil, Adefovir dipivoxil-hypoxia, Cadmium chloride, Chloroacetaldehyde, Cidofovir, Cisplatin, Clodronate, Cyclosporine A, Hypoxia, Ifosfamide, Zoladronate-hypoxia, Zoledronate.
	Human and Rat	Liver	*in vitro*	Repeat	(11)	Acetaminophen, Amiodarone, Chlorpromazine, Cyclosporine A, EMD335823, Fenofibrate, Ibuprofen, Metformin, Rosiglitazone, Troglitazone, Valproic acid.
**TG-GATEs**	Human	Liver	*in vitro*	Bolus	(160)	2-Nitrofluorene, 2,4-dinitrophenol, Acarbose, Acetamide, Acetamidofluorene, Acetaminophen, Acetazolamide, Adapin, Aflatoxin B1, Ajmaline, Allopurinol, Allyl alcohol, Alpidem, Amiodarone, Amitriptyline, Amphotericin B, Aspirin, Azathioprine, Bendazac, Benzbromarone, Benziodarone, Bromobenzene, Bromoethylamine, Bucetin, Buspirone, Buthionine sulfoximine, Butylated hydroxyanisole, Caffeine, Captopril, Carbamazepine, Carbon tetrachloride, Chloramphenicol, Chlormadinone, Chlormezanone, Chlorpheniramine, Chlorpromazine, Chlorpropamide, Cimetidine, Ciprofloxacin, Clofibrate, Clomipramine, Clozapine, Colchicine, Coumarin, Cycloheximide, Cyclophosphamide, Cyclosporine A, Danazol, Dantrolene, Dexamethasone, Diazepam, Diclofenac, Diethylmaleate, Diltiazem, Disopyramide, Disulfiram, Doxorubicin, EMD335823, Enalapril, Erythromycin, Ethambutol, Ethanol, Ethinylestradiol, Ethionamide, Ethionine, Etoposide, Famotidine, Fenofibrate, Fluoxetine, Fluphenazine, Flutamide, Furosemide, Galactosamine, Gemfibrozil, Glibenclamide, Griseofulvin, Haloperidol, Hexachlorobenzene, HGF, Hydroxyzine, Ibuprofen, IL1beta, IL6, Imipramine, Indomethacin, INFalpha, Iproniazid, Isoniazid, Ketoconazole, Labetalol, Lomustine, Lornoxicam, LPS, Mefenamic acid, Meloxicam, Metformin, Methapyrilene, Methimazole, Methyldopa, Methylene dianiline, Methyltestosterone, Mexiletine, Monocrotaline, Moxisylyte, N-methyl-N-nitrosourea, N-nitrosomorpholine, Naphthyl isothiocyanate, Naproxen, Nefazodone, Nicotinic acid, Nifedipine, Nimesulide, Nitrofurantoin, Nitrofurazone, Nitrosodiethylamine, Omeprazole, Papaverine, Pemoline, Penicillamine, Perhexiline, Phalloidin, Phenacetin, Phenobarbital, Phenylanthranilic acid, Phenylbutazone, Phenytoin, Phorone, Promethazine, Propranolol, Propylthiouracil, Quinidine, Ranitidine, Rifampicin, Rosiglitazone, Rotenone, Simvastatin, Sulfasalazine, Sulindac, Sulpiride, Tacrine, Tamoxifen, Tannic acid, Terbinafine, Tetracycline, TGFbeta1, Theophylline, Thioacetamide, Thioridazine, Ticlopidine, Tiopronin, TNF, Tolbutamide, Triazolam, Trimethadione, Troglitazone, Tunicamycin, Valproic acid, Venlafaxine, Vitamin A, WY-14643.
	Rat	Liver	*in vitro*	Bolus	(145)	Acarbose, Acetamidofluorene, Acetaminophen, Acetazolamide, Adapin, Ajmaline, Allopurinol, Allyl alcohol, Alpidem, Amiodarone, Amitriptyline, Aspirin, Azathioprine, Bendazac, Benzbromarone, Benziodarone, Bromobenzene, Bromoethylamine, Bucetin, Buspirone, Buthionine sulfoximine, Caffeine, Captopril, Carbamazepine, Carbon tetrachloride, Carboplatin, Cephalothin, Chloramphenicol, Chlormadinone, Chlormezanone, Chlorpheniramine, Chlorpromazine, Chlorpropamide, Cimetidine, Ciprofloxacin, Cisplatin, Clofibrate, Clomipramine, Clozapine, Colchicine, Coumarin, Cycloheximide, Cyclophosphamide, Cyclosporine A, Danazol, Dantrolene, Diazepam, Diclofenac, Diethylmaleate, Diltiazem, Disopyramide, Disulfiram, Doxorubicin, Enalapril, Erythromycin, Ethambutol, Ethanol, Ethinylestradiol, Ethionamide, Ethionine, Etoposide, Famotidine, Fenofibrate, Fluphenazine, Flutamide, Furosemide, Galactosamine, Gemfibrozil, Gentamicin, Glibenclamide, Griseofulvin, Haloperidol, Hexachlorobenzene, Hydroxyzine, Ibuprofen, Imipramine, Indomethacin, Iproniazid, Isoniazid, Ketoconazole, Labetalol, Lomustine, Lornoxicam, LPS, Mefenamic acid, Meloxicam, Metformin, Methapyrilene, Methimazole, Methyldopa, Methyltestosterone, Mexiletine, Monocrotaline, Moxisylyte, Naphthyl isothiocyanate, Naproxen, Nefazodone, Nicotinic acid, Nifedipine, Nimesulide, Nitrofurantoin, Nitrofurazone, Nitrosodiethylamine, Omeprazole, Papaverine, Pemoline, Penicillamine, Perhexiline, Phalloidin, Phenacetin, Phenobarbital, Phenylanthranilic acid, Phenylbutazone, Phenytoin, Phorone, Promethazine, Propylthiouracil, Puromycin, Quinidine, Ranitidine, Rifampicin, Simvastatin, Sulfasalazine, Sulindac, Sulpiride, Tacrine, Tamoxifen, Tannic acid, Terbinafine, Tetracycline, Theophylline, Thioacetamide, Thioridazine, Ticlopidine, Tiopronin, TNFalpha, Tolbutamide, Triamterene, Triazolam, Trimethadione, Tunicamycin, Valproic acid, Venlafaxine, Vitamin A, WY-14643.
	Rat	Liver	*in vivo*	Bolus	(158)	2-Nitrofluorene, 2,4-dinitrophenol, 3-Methylcholanthrene, Acarbose, Acetamidofluorene, Acetaminophen, Acetazolamide, Adapin, Aflatoxin B1, Ajmaline, Allopurinol, Allyl alcohol, Alpidem, Amiodarone, Amitriptyline, Amphotericin B, Aspirin, Azathioprine, Bendazac, Benzbromarone, Benziodarone, Bortezomib, Bromobenzene, Bromoethylamine, Bucetin, Buthionine sulfoximine, Butylated hydroxyanisole, Caffeine, Captopril, Carbamazepine, Carbon tetrachloride, Chloramphenicol, Chlormadinone, Chlormezanone, Chlorpheniramine, Chlorpromazine, Chlorpropamide, Cimetidine, Ciprofloxacin, Clofibrate, Clomipramine, Colchicine, Coumarin, Cycloheximide, Cyclophosphamide, Cyclosporine A, Danazol, Dantrolene, Dexamethasone, Diazepam, Diclofenac, Diethylmaleate, Diltiazem, Disopyramide, Disulfiram, Doxorubicin, Enalapril, Erythromycin, Ethambutol, Ethanol, Ethinylestradiol, Ethionamide, Ethionine, Etoposide, Famotidine, Fenofibrate, Fluoxetine, Fluphenazine, Flutamide, Furosemide, Galactosamine, Gefitinib, Gemfibrozil, Gentamicin, Glibenclamide, Griseofulvin, Haloperidol, Hexachlorobenzene, Hydroxyzine, Ibuprofen, Imatinib, Imipramine, Indomethacin, Iproniazid, Isoniazid, Ketoconazole, Labetalol, Lomustine, Lornoxicam, LPS, Mefenamic acid, Meloxicam, Metformin, Methapyrilene, Methimazole, Methyldopa, Methylene dianiline, Methyltestosterone, Mexiletine, Monocrotaline, Moxisylyte, N-methyl-N-nitrosourea, N-nitrosomorpholine, Naphthyl isothiocyanate, Naproxen, Nicotinic acid, Nifedipine, Nimesulide, Nitrofurantoin, Nitrofurazone, Nitrosodiethylamine, Omeprazole, Papaverine, Pemoline, Penicillamine, Perhexiline, Phalloidin, Phenacetin, Phenobarbital, Phenylanthranilic acid, Phenylbutazone, Phenytoin, Phorone, Promethazine, Propranolol, Propylthiouracil, Puromycin, Quinidine, Ranitidine, Rifampicin, Rosiglitazone, Rotenone, Simvastatin, Sulfasalazine, Sulindac, Sulpiride, Tacrine, Tamoxifen, Tannic acid, Terbinafine, Tetracycline, Theophylline, Thioacetamide, Thioridazine, Ticlopidine, Tiopronin, TNF, Tolbutamide, Triamterene, Triazolam, Trimethadione, Tunicamycin, Valproic acid, Vitamin A, WY-14643.
	Rat	Liver	*in vivo*	Repeat	(143)	2,4-dinitrophenol, Acarbose, Acetamide, Acetamidofluorene, Acetaminophen, Acetazolamide, Adapin, Ajmaline, Allopurinol, Allyl alcohol, Amiodarone, Amitriptyline, Amphotericin B, Aspirin, Azathioprine, Bendazac, Benzbromarone, Benziodarone, Bromobenzene, Bromoethylamine, Bucetin, Butylated hydroxyanisole, Caffeine, Captopril, Carbamazepine, Carbon tetrachloride, Carboplatin, Cephalothin, Chloramphenicol, Chlormadinone, Chlormezanone, Chlorpheniramine, Chlorpromazine, Chlorpropamide, Cholesterol sodium cholate 1to4, Cimetidine, Ciprofloxacin, Cisplatin, Clofibrate, Clomipramine, Colchicine, Coumarin, Cyclophosphamide, Cyclosporine A, Danazol, Dantrolene, Desmopressin acetate, Diazepam, Diclofenac, Diltiazem, Disopyramide, Disulfiram, Doxorubicin, Enalapril, Erythromycin, Ethambutol, Ethanol, Ethinylestradiol, Ethionamide, Ethionine, Etoposide,, Famotidine, Fenofibrate, Fluoxetine, Fluphenazine, Flutamide, Furosemide, Gemfibrozil, Gentamicin, Glibenclamide, Griseofulvin, Haloperidol, Hexachlorobenzene, Hydroxyzine, Ibuprofen, Imipramine, Indomethacin, Iproniazid, Isoniazid, Ketoconazole, Labetalol, Lomustine, Lornoxicam, Mefenamic acid, Meloxicam, Metformin, Methapyrilene, Methimazole, Methyldopa, Methylene, dianiline, Methyltestosterone, Mexiletine, Monocrotaline, Moxisylyte, Naphthyl isothiocyanate, Naproxen, Nicotinic acid, Nifedipine, Nimesulide, Nitrofurantoin, Nitrofurazone, Nitrosodiethylamine, Omeprazole, Papaverine, Pemoline, Penicillamine, Perhexiline, Phalloidin, Phenacetin, Phenobarbital, Phenylanthranilic acid, Phenylbutazone, Phenytoin, Promethazine, Propranolol, Propylthiouracil, Puromycin, Quinidine, Ranitidine, Rifampicin, Rosiglitazone, Rotenone, Simvastatin, Sulfasalazine, Sulindac, Sulpiride, Tacrine, Tamoxifen, Tannic acid, Terbinafine, Tetracycline, Theophylline, Thioacetamide, Thioridazine, Ticlopidine, Tiopronin, Tolbutamide, Triamterene, Triazolam, Trimethadione, Valproic acid, Vitamin A, WY-14643.
	Rat	Kidney	*in vivo*	Bolus and Repeat	(41)	Acetaminophen, Acetazolamide, Allopurinol, Allyl alcohol, Amphotericin B, Bromobenzene, Bromoethylamine, Bucetin, Caffeine, Captopril, Carboplatin, Cephalothin, Ciprofloxacin, Cisplatin, Clofibrate, Cyclophosphamide, Cyclosporine A, Desmopressin acetate, Doxorubicin, Enalapril, Erythromycin, Ethinylestradiol, Ethionine, Gentamicin, Hexachlorobenzene, Imipramine, Indomethacin, Ketoconazole, Lomustine, Methyltestosterone, Monocrotaline, Nitrofurantoin, Omeprazole, Phenacetin, Phenylanthranilic acid, Phenylbutazone, Puromycin, Rifampicin, Thioacetamide, Triamterene, Valproic acid.

Supplementary Table 3. Genes removed for lack of data for them concerning "pathway specific chemicals" in particular categories.

**Category** (Number of genes)	**Removed Genes**											
**All database** (25)	BCMO1	C12orf29	C16orf72	C8orf4	C9orf3	CBARA1	CCDC104	CCT8L1	CDC2L6	CES1	DLEU7	FAM119A
	GSTM1	GVIN1	JHDM1D	KBTBD5	LEPREL1	NME2	PION	PRR13	RBM9	RPL10A	RPL7	UGT1A1
	UGT2B7											
**Rat liver *in vitro*** *(164)**	ABCC1	ADM2	ADO	AKAP7	AKNA	AKR1B10	AKR1C2	ALDH18A1	ALDH1A3	ALDH1L2	ALKBH5	ANK2
	ASB3	B4GALNT2	B9D2	BACH1	BCMO1	BEND6	BLVRB	BTF3L4	C12orf29	C16orf72	C8orf4	C9orf3
	CABC1	CBARA1	CCDC104	CCDC109B	CCL2	CCT8L1	CD27	CDC2L6	CDH24	CDSN	CES1	CLIP4
	COCH	CORO7	CRYBG3	CTSO	CTTN	CXCL5	CYB5R1	CYP1B1	DAAM2	DENND4C	DLEU7	DLX2
	ECHS1	EDA2R	EIF1	EXOC7	FAM119A	FAM159A	FAM65C	FAM69A	FBXL2	FNDC7	FREM2	FTH1
	GLI2	GPATCH3	GSTA1	GSTA4	GSTM1	GSTM3	GTPBP4	GVIN1	H2AFZ	HAX1	HDAC8	HIST1H1C
	HMHB1	HRASLS2	IDS	IFT172	INPP5B	INSIG1	IRF4	JHDM1D	KBTBD5	KCNT2	LAYN	LEPREL1
	LONP1	LPL	LTA	MAGOHB	MAMDC2	MAPK8	MAZ	MEGF9	MFF	MSC	MT2A	NCL
	NME2	NOS2	NPNT	NRBF2	NUPR1	OIT3	PAQR3	PDDC1	PGAP1	PHF10	PION	PLA2G4A
	PMAIP1	POP5	PPP2R5A	PRDX1	PRR13	PRSS35	PSG5	PTBP1	PTGS2	RAB39B	RBM39	RBM9
	RPL10A	RPL13A	RPL7	RPRD2	RPS6	RUNX2	RXRA	SDCCAG8	SH3RF2	SLC16A14	SLC1A4	SLC1A7
	SLC2A11	SLC44A3	SLMAP	SLTM	SNAI2	SNRNP35	SNX1	SNX22	SOAT2	SPEN	SURF6	TARS
	TLCD2	TMEFF2	TMEM74	TMTC2	TPX2	TRIM69	TSLP	UBE2L3	UBE2V2	UBE2W	UGT1A1	UGT1A6
	UGT2B7	VIPR1	VNN3	WDR27	WDR63	YLPM1	ZFC3H1	ZNF268				
**Rat liver *in vivo*** *(196)***	ABCC1	ACAP2	ADAM23	ADCY1	ADM2	ADO	AFF1	AGPAT9	AKAP7	AKNA	AKR1B10	AKR1C2
	ALDH18A1	ALDH1A3	ALDH1L2	ALKBH5	ALS2	ANK2	ASB3	B4GALNT2	B9D2	BACH1	BCMO1	BEND6
	BLVRB	BTF3L4	C12orf29	C16orf72	C8orf4	C9orf3	CASC5	CBARA1	CCDC104	CCL2	CCT8L1	CD27
	CDC2L6	CDH24	CDSN	CES1	CLIP4	COCH	COL24A1	CRYBG3	CTSO	CTTN	CYB5R1	CYP1B1
	DAAM2	DENND4C	DLEU7	DLX2	DUT	ECHS1	EDA2R	EDEM3	EIF1	ELF4	EXOC7	FAM119A
	FAM159A	FAM65C	FAM69A	FBXL2	FLRT1	FNDC7	FREM2	FTH1	GLI2	GPATCH3	GSK3A	GSTA1
	GSTA4	GSTM1	GSTM3	GTPBP4	GVIN1	H2AFZ	HAX1	HDAC8	HIST1H1C	HMHB1	HRASLS2	IDH1
	IDS	IFT172	INPP5B	INSIG1	IRF4	JHDM1D	KBTBD5	KCNT2	KIF13B	KLHDC10	KRAS	LAYN
	LEPREL1	LONP1	LPL	LRRK1	MAGOHB	MAMDC2	MANEA	MAPK8	MAZ	MEGF9	MFF	MSC
	MT2A	MTM1	NCL	NME2	NOS2	NPNT	NRBF2	NREP	NUPR1	OIT3	ORMDL3	OSMR
	PAQR3	PCDH7	PDDC1	PGAP1	PHF10	PION	PLA2G4A	PMAIP1	POP5	PPP2R5A	PRDX1	PRR13
	PRSS35	PSG5	PTBP1	PTGS2	PYCR1	RAB39B	RBM39	RBM9	RPL10A	RPL13A	RPL7	RPRD2
	RPS6	RUNX2	RXRA	SDCCAG8	SEMA3E	SH3RF2	SLC16A14	SLC16A9	SLC1A4	SLC1A7	SLC2A11	SLC44A3
	SLMAP	SLTM	SNAI2	SNRNP35	SNX1	SNX22	SOAT2	SPEN	SPRED1	SRPK1	SUB1	SURF6
	TAB2	TARS	TBCEL	TLCD2	TMEFF2	TMEM154	TMEM74	TMTC2	TNKS	TPX2	TRIM69	TSLP
	UBE2L3	UBE2V2	UBE2W	UGT1A1	UGT1A6	UGT2B7	VIPR1	VNN3	WDR27	WDR63	YLPM1	ZBTB38
	ZC3HAV1	ZDHHC20	ZFC3H1	ZNF268								
**Human liver *in vitro*** *(44)****	ACOT2	AKAP2	AMACR	ANAPC1	ATF4	BCMO1	BGLAP	C12orf29	C16orf72	C8orf4	C9orf3	CABC1
	CBARA1	CCDC104	CCT8L1	CD302	CDC2L6	CES1	DLEU7	EIF3C	FAM119A	FAM188B	FBF1	GSTM1
	GSTM2	GVIN1	HHIPL1	JHDM1D	KBTBD5	LEPREL1	NME2	PAIP1	PION	PRR13	PTCD1	RAN
	RBM9	RFFL	RPL10A	RPL7	TMIGD1	TXNDC5	UGT1A1	UGT2B7				

* *All genes that were removed from the "Rat liver in vitro" category were removed from "Rat liver in vivo" category as well except five:* ***CABC1*****,** ***CCDC109B*****,** ***CORO7*****,** ***CXCL5*** *and* ***LTA***.

** *All genes that were removed from the "Rat liver in vivo" category were removed from "Rat liver in vitro" category as well except 37:* **ACAP2**, **ADAM23**, **ADCY1**, **AFF1**, **AGPAT9**, **ALS2**, **CASC5**, **COL24A1**, **DUT**, **EDEM3**, **ELF4**, **FLRT1**, **GSK3A**, **IDH1**, **KIF13B**, **KLHDC10**, **KRAS**, **LRRK1**, **MANEA**, **MTM1**, **NREP**, **ORMDL3**, **OSMR**, **PCDH7**, **PYCR1**, **SEMA3E**, **SLC16A9**, **SPRED1**, **SRPK1**, **SUB1**, **TAB2**, **TBCEL**, **TMEM154**, **TNKS**, **ZBTB38**, **ZC3HAV1** *and* ***ZDHHC20***.

****All genes that were had no rat data (in vitro or in vivo) had no human data neither (exception:* ***CABC1*** *removed from "Rat Liver in vivo" but not from human data), but the opposite is not always true: 18 of the genes that were removed for lack of human data, were kept for both rat categories (****ACOT2, AKAP2, AMACR, ANAPC1, ATF4, BGLAP, CD302, EIF3C, FAM188B, FBF1, GSTM2, HHIPL1, PAIP1, PTCD1, RAN, RFFL, TMIGD1*** *and* ***TXNDC5****).*

Supplementary Table 4. Annotation of compounds for figure 5, 6 and 7.

Nb	Chemical	Nb	Chemical	Nb	Chemical	Nb	Chemical
1	2-Nitrofluorene	41	Clomipramine	81	Ibuprofen	121	Phalloidin
2	2-4-dinitrophenol	42	Clozapine	82	IL1beta	122	Phenacetin
3	Acarbose	43	Colchicine	83	IL6	123	Phenobarbital
4	Acetamide	44	Coumarin	84	Imipramine	124	Phenylanthranilicacid
5	Acetamidofluorene	45	Cycloheximide	85	Indomethacin	125	Phenylbutazone
6	Acetaminophen	46	Cyclophosphamide	86	INFalpha	126	Phenytoin
7	Acetazolamide	47	Cyclosporine A	87	Iproniazid	127	**Phorone**
8	Adapin	48	Danazol	88	Isoniazid	128	Promethazine
9	Aflatoxin B1	49	Dantrolene	89	Ketoconazole	129	Propranolol
10	Ajmaline	50	Dexamethasone	90	Labetalol	130	Propylthiouracil
11	Allopurinol	51	Diazepam	91	Lomustine	131	Quinidine
12	Allyl alcohol	52	Diclofenac	92	Lornoxicam	132	Ranitidine
13	Alpidem	53	Diethylmaleate	93	LPS	133	Rifampicin
14	Amiodarone	54	Diltiazem	94	Mefenamic acid	134	Rosiglitazone
15	Amitriptyline	55	Disopyramide	95	Meloxicam	135	Rotenone
16	Amphotericin B	56	Disulfiram	96	Metformin	136	Simvastatin
17	Aspirin	57	Doxorubicin	97	Methapyrilene	137	Sulfasalazine
18	Azathioprine	58	EMD335823	98	Methimazole	138	Sulindac

**19**	Bendazac	**59**	Enalapril	**99**	Methyldopa	**139**	Sulpiride
**20**	Benzbromarone	**60**	Erythromycin	**100**	Methylene dianiline	**140**	Tacrine
**21**	Benziodarone	**61**	Ethambutol	**101**	Methyltestosterone	**141**	Tamoxifen
**22**	Bromobenzene	**62**	Ethanol	**102**	Mexiletine	**142**	Tannic_acid
**23**	Bromoethylamine	**63**	Ethinylestradiol	**103**	Monocrotaline	**143**	Terbinafine
**24**	Bucetin	**64**	Ethionamide	**104**	Moxisylyte	**144**	Tetracycline
**25**	Buspirone	**65**	Ethionine	**105**	N-methyl-N-nitrosourea	**145**	TGFbeta1
**26**	Buthionine sulfoximine	**66**	Etoposide	**106**	N-nitrosomorpholine	**146**	Theophylline
**27**	Butylated hydroxyanisole	**67**	Famotidine	**107**	Naphthyl isothiocyanate	**147**	Thioacetamide
**28**	Caffeine	**68**	Fenofibrate	**108**	Naproxen	**148**	Thioridazine
**29**	Captopril	**69**	Fluoxetine	**109**	Nefazodone	**149**	Ticlopidine
**30**	Carbamazepine	**70**	Fluphenazine	**110**	Nicotinic_acid	**150**	Tiopronin
**31**	Carbon tetrachloride	**71**	Flutamide	**111**	Nifedipine	**151**	TNF
**32**	Chloramphenicol	**72**	Furosemide	**112**	Nimesulide	**152**	Tolbutamide
**33**	Chlormadinone	**73**	Galactosamine	**113**	Nitrofurantoin	**153**	Triazolam
**34**	Chlormezanone	**74**	Gemfibrozil	**114**	Nitrofurazone	**154**	Trimethadione
**35**	Chlorpheniramine	**75**	Glibenclamide	**115**	Nitrosodiethylamine	**155**	Troglitazone
**36**	Chlorpromazine	**76**	Griseofulvin	**116**	**Omeprazole**	**156**	**Tunicamycin**
**37**	Chlorpropamide	**77**	Haloperidol	**117**	Papaverine	**157**	Valproic acid
**38**	Cimetidine	**78**	Hexachlorobenzene	**118**	Pemoline	**158**	Venlafaxine
**39**	Ciprofloxacin	**79**	HGF	**119**	Penicillamine	**159**	Vitamin A
**40**	Clofibrate	**80**	Hydroxyzine	**120**	Perhexiline	**160**	WY-14643

2 Supplementary Figures

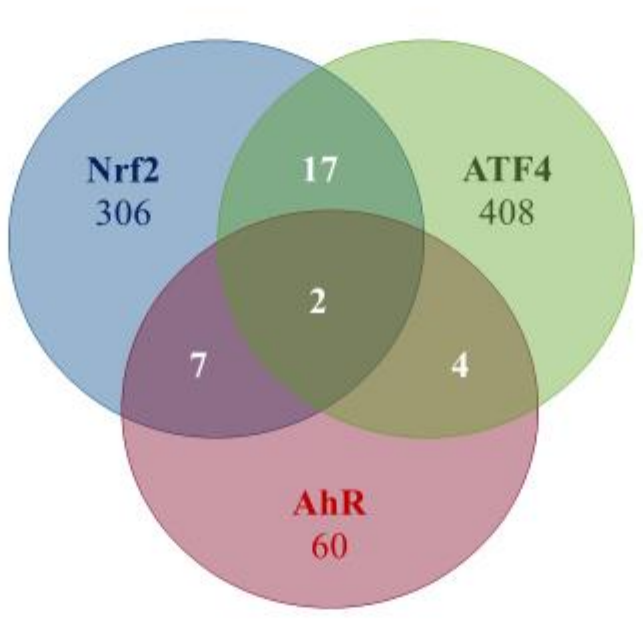

Supplementary Figure 1. Venn diagram showing the number of genes found in each of the three pathways AhR, Nrf2 and ATF4.
